# Personalized LSTM Models for ECG Lead Transformations Led to Fewer Diagnostic Errors Than Generalized Models: Deriving 12-Lead ECG from Lead II, V2, and V6

**DOI:** 10.3390/s23031389

**Published:** 2023-01-26

**Authors:** Prashanth Shyam Kumar, Mouli Ramasamy, Kamala Ramya Kallur, Pratyush Rai, Vijay K. Varadan

**Affiliations:** 1 The Department of Engineering Science and Mechanics, The Pennsylvania State University, 212 Earth-Engineering Sciences Bldg, University Park, PA 16802, USA; 2 Geisinger Medical Center, 100 North Academy Avenue, Danville, PA 17822, USA; 3 The Department of Biomedical Engineering, The University of Arkansas, 4183 Bell Engineering Center, Fayetteville, AR 72701, USA; 4 The Department of Neurosurgery, Milton S. Hershey Medical Center, 500 University Dr, Hershey, PA 17033, USA

**Keywords:** ECG, LSTM networks, Bayesian Optimization, personalized medicine, wearable devices

## Abstract

Background and Objective: The prevalence of chronic cardiovascular diseases (CVDs) has risen globally, nearly doubling from 1990 to 2019. ECG is a simple, non-invasive measurement that can help identify CVDs at an early and treatable stage. A multi-lead ECG, up to 15 leads in a wearable form factor, is desirable. We seek to derive multiple ECG leads from a select subset of leads so that the number of electrodes can be reduced in line with a patient-friendly wearable device. We further compare personalized derivations to generalized derivations. Methods: Long-Short Term Memory (LSTM) networks using Lead II, V2, and V6 as input are trained to obtain generalized models using Bayesian Optimization for hyperparameter tuning for all patients and personalized models for each patient by applying transfer learning to the generalized models. We compare quantitatively using error metrics Root Mean Square Error (RMSE), R^2^, and Pearson correlation (ρ). We compare qualitatively by matching ECG interpretations of board-certified cardiologists. Results: ECG interpretations from personalized models, when corrected for an intra-observer variance, were identical to the original ECGs, whereas generalized models led to errors. Mean performance values for generalized and personalized models were (RMSE-74.31 µV, R^2^-72.05, ρ-0.88) and (RMSE-26.27 µV, R^2^-96.38, ρ-0.98), respectively. Conclusions: Diagnostic accuracy based on derived ECG is the most critical validation of ECG derivation methods. Personalized transformation should be sought to derive ECGs. Performing a personalized calibration step to wearable ECG systems and LSTM networks could yield ambulatory 15-lead ECGs with accuracy comparable to clinical ECGs.

## 1. Introduction

Globally, the leading causes of mortality and disability are ischemic heart disease and stroke [1]. The prevalence and mortality of cardiovascular diseases (CVD) have increased from 271 million to 523 million and 12.1 million to 18.6 million, respectively, from 1990 to 2019. In the U.S., a conservative projection by Pearson-Stuttard et al. [2] estimated that total coronary and stroke deaths by 2030 will increase by ≈18% and 50%, respectively. Total costs (direct and indirect costs) of CVD were estimated to be USD 555 billion in 2015. These costs are expected to double to USD 1.1 trillion by 2035. The looming shortages of trained physicians further complicate the increased burden of CVDs. The U.S. could have an estimated shortage of 54,100 to 139,000 physicians by 2033 [3]. These shortages may continue to grow as the population ages. More Americans live longer with chronic diseases and require longitudinal care.

The current era of digital health may provide a means to lessen the burden of the reduced physician-to-patient ratio. eHealth (electronic Health) and mHealth (mobile Health) have been extensive research topics over the past two decades. In 2016 alone, global smartphone sales reached close to 1.5 billion, one for every fifth person on earth [4]. There are several consumer devices by companies such as Apple Inc. (Cupertino, CA, USA), Fitbit (currently owned by Google Inc., Mountain View, CA, USA), and Samsung in the market with form factors such as wrist-worn, ring, and necklace-styles that can collect physiological data such as heart rate and photoplethysmography. Data from the Apple Watch device have been used to detect atrial fibrillation [5]. However, they must be used with care [6].

There is a critical need for devices including software decision support tools that are non-inferior to traditional medical devices used in hospital settings. Advances in this area will play a key role in boosting healthcare providers’ capacity to meet the projected CVD management needs.

The standard clinical version of the ECG is the 12-lead ECG consisting of Lead I, II, and III, which are bipolar, aVR, aVL, and aVF, which are augmented unipolar, and V1 through V6, which are unipolar. This system requires the placement of 10 electrodes on the patient’s skin. Vectorcardiography (VCG) [7] is an essential complement to the standard 12-lead (S12) ECG. It is a three-dimensional representation of the cardiac vector loop in three orthogonal planes: vertical, transverse, and sagittal. VCG is less often used in a clinical setting than S12. However, the VCG adds diagnostic value in several conditions that complement the S12 [8,9].

Long-term Monitoring (LTM) utilizes a class of devices that consist of single to multi-lead adhesive patch integrated devices, Holter monitors, event recorders, and implantable loop recorders. They are used for ECG monitoring while the patient is ambulatory and have a reduced set of leads ranging from a single-lead patch to a seven-lead recorder. Many current devices have wireless connectivity and upload event-related data in real-time. LTM is primarily used for the detection of transient rhythm abnormalities such as atrial fibrillation, premature ventricular contractions (PVCs), pauses and tachy-and bradycardia, and they have been proven to be more effective compared to Short-term Monitoring (STM) for this purpose [10,11]. STM predominantly utilizes standard bedside medical equipment to record the standard 12-lead ECG. The patient is stationary and usually supine during the recording period, and recording lengths range from 10 to 30 s. STM is more effective in determining persistent pathological conditions with a high specificity level than LTM. 

While STM predominantly uses traditional bedside monitors, LTM has seen several advances in recent decades. The devices that have proven to be most effective have an internet-connected architecture [12]. Among the several devices that have been proposed in the literature, the following ECG monitoring devices have clearance from FDA in the U.S. and have achieved significant clinical adoption—NUVANT MCT [13], Zio Patch [14], and Kardia Mobile [15].

From a clinical perspective, the trends toward remote monitoring and diagnostics powered by digital health have created a greater demand for tools with high diagnostic value for home use. These tools must be comparable to the tools used in hospitals. In many cases, data from devices such as wearable ECGs with higher specificity and sensitivity can help save time and costs of hospital visits and minimize the number of tests required to arrive at a diagnosis. This is evidenced by the number of commercial devices used as the standard of care as described earlier. The standard 12-lead (S12) is still the gold standard for diagnosis, so a blend of the diagnostic specificity of the multi-lead STM and the sensitivity of long recording durations of the LTM is desired. Notably, the putatively best-performing Artificial Intelligence-based methods for diagnostics based on ECG require S12 as the input to achieve high performance [16,17,18,19,20,21]. 

From an engineering perspective, the following constraints exist for wearable ECG device designs: First, standard lead systems have electrodes placed far apart. The greater the separation between electrodes, the more noise is likely introduced due to motion. All electrodes or sensors need to be electrically connected to electronics, so the wearable device must cover most of the body when sensors are far apart. Second, for remote monitoring, the quality of data connection or connection to the internet must be adequate to support data transfer at higher volumes, or more complex compression algorithms are needed, increasing the computing requirements on the device and the power requirements. Third, as the number of leads recorded increases:

- More power is needed, i.e., a larger sized and higher capacity battery to:
  ○ Acquire, condition, and store the data on the wearable device. More channels of Analog to Digital Conversion would be needed and the amount of energy needed to write additional data to onboard memory on the wearable device will also increase [22].
  ○ Transfer the data to a smart device or data gateway device.
- More storage is needed to archive the data that is uploaded to the cloud. Cloud storage increases in cost if retained for long periods.
- More electrodes need to be placed on the skin, making the device cumbersome.

**Figure 1:**
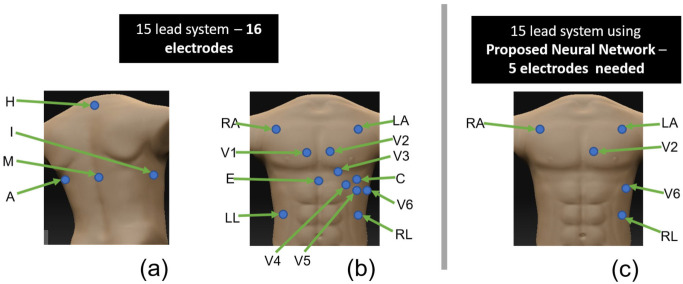
Comparison of electrode placement needed for a 15-lead system and the proposed method. For 15-lead system (**a**) electrode placements on the ventral side (**b**) electrode placements on the dorsal side. For the proposed method (**c**) electrode placements only on the ventral side [7,23].

These constraints dictate that wearable ECG monitors should be designed with a minimal number of electrodes and a minimal number of leads. A multi-lead ECG, ideally all 15 leads in a wearable form factor, is highly desirable in the new digital health era. The number of electrodes that need to be placed on the skin to acquire these ECGs is depicted in Figure 1. The S12 requires 10 electrodes, while (VCG) requires 7 electrodes. Only one electrode location, i.e., left leg, is shared between these lead systems. Ideally, if we wanted all 15 leads in an ECG measurement system, we would need 16 electrodes placed on the patient. However, based on the constraints described, the constraint for wearable ECG devices stems from the number of electrodes required to provide all the clinical information necessary to unlock the diagnostic power of a multi-lead ECG system. It is impractical to have 16 electrodes or sensors placed at a precise anatomic location to obtain clinical-grade ECGs. It is also impractical to collect, store, and transfer large amounts of data per patient.

A method to compute multiple leads from a reduced lead system is desired to obtain the combined benefit of LTM and STM. This task, in essence, is a function approximation task. The function transforms a reduced set of leads into a larger set of leads. Therefore, a method is presented to reduce the number of electrodes needed so that a wearable device that captures ECG can be designed to capture all information needed for an accurate diagnosis without compromising the quality of life for patients and diagnostic utility. We present this method as a complementary technique to wearable ECG monitoring technology that our research group has previously demonstrated using cloth-based Nanosensor technology [24]. Even though the electrode placements in the proposed method span the whole area of the chest, a wearable device in a textile form factor can be designed to capture the necessary leads with the advantage of not requiring adhesives, conductive gels, or skin preparation [25]. This paper makes the following original contributions to the body of knowledge:

- The existing literature does not describe or extensively characterize a methodology to transform a reduced set of ECG leads into a complete set of leads, including Frank XYZ vectorcardiography using an LSTM neural network. A novel deep neural network approach and a detailed validation strategy for the appropriate choice of hyperparameters using Bayesian global optimization are presented.
- We propose a transfer learning approach to create personalized models for each patient so that the ECG transformations can account for each individual’s unique anatomy. The personalized models were the most accurate based on quantitative and qualitative assessments.

## 2. Related Work

Several of the initial research efforts on the transformation of ECGs focused on transformations from S12 to Frank XYZ so that clinicians can tap into the added specificity and sensitivity of VCGs while following the standard of care, which only requires the measurement of the standard 12-lead ECG. From 1986 to 2009, researchers used linear regression to approximate the transformation function. Table 1 lists the ECG transformation studies reported in the literature. The accuracy of lead transformations to Frank XYZ could be reproduced from several results reported in the literature (Figures S12–S14 in the Supplementary Material). Among these results, we chose the inputs to be Lead II, V2, and V6 under the assumption that they showed a good performance in terms of errors, and the leads were quasi-orthogonal [26], which could imply that they carry the maximal information needed to reconstruct the remaining leads.

In 2010, the first neural network-based transformation was proposed [27]. Since then, researchers have made several efforts to address the practical challenge of reducing leads acquired while maintaining diagnostic yield. Most studies focus on using a three-lead ECG as the input to a transformation that will output 12 lead ECG. Several studies have used closed datasets explicitly acquired for the research and are now unavailable for other researchers. A few studies used open databases, such as the Physionet data bank [28]. One open dataset that is ideally suited for this research is the PTB diagnostic ECG repository. 

Root mean square (RMS) and pearson correlation coefficient are the most reported metrics. R squared, defined as in (3), is used in the literature. Therefore, the following metrics form the most detailed evaluation: RMS error, pearson correlation coefficient, and R^2^. There is a fundamental limitation to the proposed techniques from 1986 to 2009, which assumed linearity so that the cardiac vector could be projected to the skin to obtain ECG waveforms. The projection of the cardiac vector assumes that the transformation of the electrical activity of the cardiac vector to the surface of the body is a strictly linear operation, which is not true as the human body has various organs and tissue between the heart and the skin with different electrical properties that will effectively result in an arbitrarily complex transformation.

**Table 1:** Related work in the literature that proposes lead transformations.

Source Lead → Target Lead	Study Population/Transformation Method	Reported Performance Metrics
S12 → Frank XYZ [29]	41 patients (closed)/Linear regression	QRS, ST and T amplitudes
S12 → Frank XYZ [30]	39 normal, 41 patients/Linear regression	R wave amplitudes
S12 → Frank XYZ [26]	Development Set 147 (30% normal, 15% hypertrophy, 30% MI, 25% other), test set 90 (30% normal, 25% hypertrophy, 30% MI, 15% other) (closed)/Linear regression	$\mathrm{Distance}\;\mathrm{Measure}\;D=\frac{1}{K}\sum_{k=1}^{K}\frac{\left|V_{k}-V_{k}^{*}\right|}{\left|V_{k}\right|}$
S12 → Frank XYZ [31]	Total 346 cases (closed)/Linear regression	Pearson Correlation coefficient
S12 → Frank XYZ [32]	PTB diagnostic ECG database excluding atrial arrhythmias or A.V. block and patients with implanted Pacemakers. (open)/Linear Regression	RMS error; Pearson Correlation coefficient
S12 → Frank XYZ [33]	PTB diagnostic ECG database only healthy and post- MI included (open)/Linear Regression	R^2^
Lead I, II and V2 → S12 [27]	120 patients (closed)/Neural Network and Linear Regression	RMS error; Pearson Correlation coefficient
Three bipolar leads→ S12 [34]	30 normal, 35 patients (closed)/Linear Regression	RMS error; Pearson Correlation coefficient
Three bipolar leads→ S12 [35]	20 normal, 22 patients(closed)/Regression Trees	Pearson Correlation coefficient
Lead I, II, and V2 → S12 [36]	39 patients were randomly chosen from PTB diagnostic ECG database (open)/Linear Regression	RMS error; Pearson Correlation coefficient
Lead I, II, and V2 → S12 [37]	39 patients were randomly chosen from PTB diagnostic ECG database (open)/LSTM neural network	RMS error; Pearson Correlation coefficient
Three bipolar leads→ S12 [38]	14 normal(closed)/Neural Network and linear regression	Pearson Correlation coefficient
Three bipolar leads→ S12 [39]	30 normal, 30 patients(closed)/LSTM neural network	RMS error; Pearson Correlation coefficient
This work—Lead II, V2, and V6 → S12 lead and Frank XYZ	PTB diagnostic ECG all records except three that are corrupted with too much noise. (open)	RMS error; Pearson Correlation coefficient, R^2^

Therefore, the goal is to arrive at an arbitrarily complex function that transforms a subset of ECG leads into a larger set of leads. Neural networks are ideally suited for such arbitrary function approximation tasks.

## 3. Materials and Methods

We implemented all data analysis programs and applications on MATLAB 2021a Update 5 version 9.10.0.1739362 (MathWorks Inc., Natick, MA, USA). The hardware consisted of an Intel processor (i7-7820X), 32 GB of RAM, and an NVIDIA RTX 3090 Graphics Processing Unit (GPU). Since the data used in this study were publicly available, the study was exempt from IRB approval by the Office for Research Protections at the Pennsylvania State University. 

### 3.1. Data Sources and Preparation

The PTB database [40] includes 15 lead ECGs from 249 patients. In some patients, multiple recordings are included so that the total number of ECGs is 549. The ECGs are sampled at 1 kHz. Only one diagnosis is included per patient in this dataset. Notably, patients will usually have several comorbidities. Myocardial Infarction (MI) patients and healthy controls account for the majority. Three recordings were rejected from further processing:

- patient095—record number 291—No V1 lead recording
- patient285—record number 537—Completely corrupted with no visible ECG data
- patient220—record number 453—No lead III data

All 549 recordings were bandpass filtered with a passband of 0.05 Hz to 45 Hz. This passband is acceptable according to long-term monitoring standards. Furthermore, we down sampled the data from 1000 Hz to 200 Hz. Firstly, for adults, most of the ECG signal content is below 100 Hz [41]. Secondly, the lower sampling rate reduces the amount of data per iteration while training neural networks.

### 3.2. Preparation of Patient-Specific Training Data for Personalized Models

Some patients have several recordings at different times, whereas others only have one recording. A sliding window data augmentation strategy was followed for each recording to increase the number of training samples available per patient. The window size was set to 17 s, and the overlap was 16 s. This sliding window data augmentation approach was followed in similar related work [39].

### 3.3. Transformation Performance Evaluation

All evaluations and measurements of performance are only calculated on the validation dataset for all methods. This is carried out to avoid bias due to expected higher performance, i.e., lower RMSE, of the neural network methods on the training data. We split the complete data set with uniform randomization into 80%/20% (training/testing). We computed the performance of the transformation only on the testing data set for all transformation methods for an unbiased comparison of performance. As mentioned earlier in the related work section, we computed the following metrics: RMS error, pearson correlation coefficient, and R^2^. The definitions of the metrics are as follows:

(1) $$RMSE=\sqrt{\frac{\sum_{i=1}^{N}{\left(y\left[i\right]-\hat{y}\left[i\right]\right)}^{2}}{N}}\;$$

(2) $$Pearson\;Correlation\;Coefficient=\left\{\frac{\sum_{i=1}^{N}\hat{y}\;\left[i\right]*y\;\left[i\right]\;}{{\left(\sum_{i=1}^{N}y\;{\left[i\right]}^{2}*\sum_{i=1}^{N}\hat{y}\;{\left[i\right]}^{2}\right)}^{\frac{1}{2}}}\right\}\;$$

(3) $$R^{2}=\left\{1-\frac{\sum_{i=1}^{N}\;{\left[\hat{y}\;\left[i\right]-y\;\left[i\right]\right]}^{2}}{\sum_{i=1}^{N}\;{\left[y\;\left[i\right]\right]}^{2}}\right\}*100$$

where N is the length of the ECG segments in samples, y is the actual measured ECG, and $\hat{y}$ is the derived ECG.

### 3.4. Transformation Performance Evaluation

We used a neural network that we believe is well-suited for time-series data, including the ECG, specifically the Long-Short-term Memory (LSTM) network. The LSTM architecture was proposed in 1997 by Hochreiter and Schmidhuber [39]. Greff et al. have performed a comprehensive search through several variants of the LSTM architecture to find that there is no significant improvement over the original LSTM architecture [40], so the original LSTM architecture is used in this research. In this work, we trained a deep learning model to learn a transfer function to derive a set of ECG leads from a different set of ECG leads. Since this is a regression type of problem that falls under the category of sequence-to-sequence translation, the loss function or cost function is half mean-square without normalization for the number of output dimensions (4); in this case, channels of ECG that are estimated. Adam optimizer [42] was chosen for the rule to apply the weight updates.

(4) $$loss=\frac{1}{2S}\sum_{i=1}^{S}\sum_{j=1}^{R}{\left({\hat{y}}_{ij}-y_{ij}\right)}^{2}$$

where S is the length of the sequence or number of samples of ECG, R is the number of channels of ECG at the output of the network, $\hat{y}$ is the estimated output at an instant of time, and y is the observed sample of ECG at that instant of time. The input weights were initialized with glorot initialization [43], where the weights were independently sampled from a uniform distribution with mean=0 and

(5) $$variance=2/\left(\left(Input\;Size+4*number\;of\;hidden\;units\right)\right)$$

The recurrent weights were initialized as Q, the result of Q.R. decomposition of a random matrix sampled from a unit normal distribution [44]. The forget gate biases were initialized with ones, and zeros were used for the remaining gates. 

The following is a list of hyperparameters whose values need to be defined to finalize a network architecture before training.

- Number of layers.
- Number of hidden units per layer.
- Learning rate.
- Minibatch Size (number of training samples per iteration)
- Learning rate schedule whether no changes or change rules for the learning rate as training progresses. The learning rate can be reduced as training progresses to allow more refined tuning of the network weights closer as the cost function reaches the global minimum.
- Adam optimizer parameters:
- $\beta_{1}$ —momentum coefficient.
- $\beta_{2}$—RMS prop coefficient.

These hyperparameter values influence the performance obtained from the networks in terms of error (RMSE, R^2^, and pearson correlation coefficient). A grid search is a deterministic method of obtaining the global minimum that a particular set of hyperparameter choices can define. All possible permutations of hyperparameters are used to train several neural networks, and the network that yields the lowest error can then be chosen. Consequently, this network would have the ideal choices for hyperparameters. However, this is a brute force method that is impractical when evaluating computationally expensive functions such as the training of a multilayer neural network. Alternatively, one might randomly choose and evaluate sets of hyperparameters, but this method may not be reproducible and could lead to optimal results only by chance. A superior approach is to use a guided search method in the space of hyperparameters. Bayesian optimization (BOpt) is an approach that is best suited for computationally expensive functions [45].

The 546 usable records were sequestered into training and testing with an approximate ratio of 80/20. The number of records in the training set was 437, and the testing was 109. All networks were trained for 100 epochs. The number of layers was not part of the hyperparameter exploration experiments described. We chose to evaluate the best performance across multiple layered networks to understand the impact of additional layers on the optimal performance found through BOpt. Therefore, hyperparameter tuning was conducted for 1-, 2-, 3-, 4-, and 5-layer networks independently, and results were compared thereafter to determine the impact of the number of layers of LSTM on the best performance achievable.

### 3.5. Hyperparameter Tuning Using Bayesian Optimization

BOpt is utilized to obtain optimal values for the hyperparameters of the LSTM network. Table 2 provides the stepwise description of the algorithm for BOpt.

The method of applying BOpt involves three key elements:

- A Gaussian Process Model (Q(f|x, y)), where $f\left(x\right)$ is the objective function defined as the final validation RMSE for a network trained with the hyperparameters defined in x, and y is the value of this RMSE. The model uses the kernel function ARD Matérn 5/2.

(6) $$k_{ARD\;Matern\frac{5}{2}}(x_{i},x_{i}|\theta )=\sigma_{f}^{2}\left(1+\sqrt{5}r+\frac{5}{3}r^{2}\right)\exp(-\sqrt{5}r)\;$$

where $r=\sqrt{\overset{d}{\underset{m=1}{\sum }}\frac{{\left(x_{im}-x_{jm}\right)}^{2}}{\sigma_{m}^{2}}}$, $x_{i}$ and $x_{j}$ are vectors of length d.

- An update procedure for (Q(f|x, y)) upon each new evaluation.
- An acquisition function $a\left(x\right)$ that is based on (Q(f|x, y)) that is maximized so that the next evaluation point x can be chosen. The choice of $a\left(x\right)$ was expected improvement [46].

**Table 2:** Description of Bayesian optimization algorithm used for hyperparameter tuning (pseudocode included in Supplementary Material).

Setup	Set the bounded range of values that each hyperparameter can assume. Set the sampling probability transformation that should be applied to the range of values (Logarithmic-scaled or Uniform). Set the limit for the total number of evaluations of the neural network training function as a stopping criterion.
Initialization	$\mathrm{Evaluate}\;f\left(x\right)$ for neural network architectures defined with four randomly sampled sets of hyperparameters from the transformed and bounded range of hyperparameters. Obtain an initial Q(f|x, y)
Iteration (while total number of *f*(*x*) evaluations < 50)	Find a new x for evaluation that maximizes the function $a\left(x\right)$ Update Q(f|x, y) after computing $f\left(x\right)$ for the new point x
Stopping	Return the best result as set of hyperparameters associated with the lowest Final Validation RMSE.

(7) $$\left(Expected\;Improvement\right)EI\left(x,\;Q\right)=E_{Q}\;\left[\max\left(0,\;\mu_{Q}\left(x_{Optimal}\right)-f\left(x\right)\right)\right]$$

where $\mu_{Q}\left(x_{Optimal}\right)$ is the lowest value of the posterior mean and $x_{Optimal}$ is the location in hyperparameter space of the lowest posterior mean. In addition to this choice of $a\left(x\right)$, another criterion was applied to increase the propensity for sampling x and avoid overexploitation of more granular sampling within a local minimum of x. This is implemented as a further constraint in the selection of the next x to evaluate. x is chosen as the next point to evaluate if the following criterion is met:

(8) $$\sigma_{f\left(x\right)}\geq 0.8*\sigma$$

where $\sigma_{f\left(x\right)}$ is the standard deviation of the posterior objective function at x, and σ is the posterior standard deviation of the additive noise. Table 3 lists the bounded range of values for each hyperparameter and the sampling transformations.

### 3.6. Personalized Network Training

The optimal network architectures that are chosen through the hyperparameter tuning process described in the previous section (Section 3.5) can be trained further with data from each patient to obtain networks that are specific for each patient. This approach of further training pre-trained networks with more specific data falls under a class of techniques for problem-solving using machine learning called transfer learning [47]. The advantage of this approach is that the amount of data available from one patient can be small. The learned weights from the general model provide an optimal starting point from which the training of a personalized model can result in an accurate model with fewer data. In the data set used for this work, there are 549 recordings across 290 patients, an average of 200 s per recording, and some patients may have only 100 s of data. Using all the patients’ data to obtain a general model and then further training the model with data from a specific patient alleviates the need for long recordings from each patient to train an accurate model.

The network architectures, including hyperparameter values and the weights, were inherited from the optimal models found through BOpt. Each network was trained for 100 epochs with the augmented data described in Section 3.2. The loss function was the same as the generalized models (4). We split the augmented data into 80/20 sets for training and validation, similar to the general networks described in Section 3.3.

### 3.7. Blinded Assessment for Qualitative Comparison

**Figure 2:**
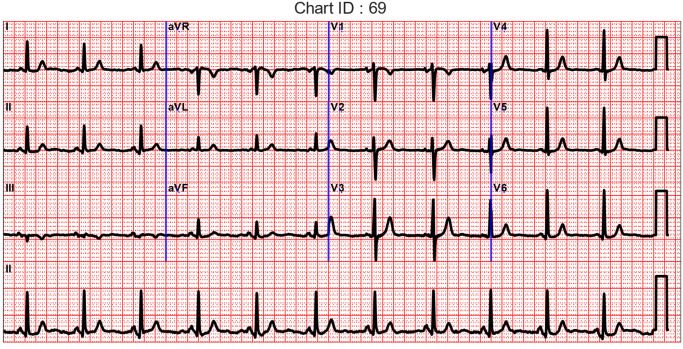
Sample of the ECG chart presented to cardiologists blinded to the source of the waveform, whether actual or derived from a subset of ECG leads.

Clinically, the ECGs derived through personalized models and the original ECG data should result in the same diagnosis. Twenty patients from the validation data set were chosen, and their respective actual ECG data was plotted with the conventional grid lines indicating time and amplitude (vertical amplitude grid 10 mm = 1 mV; horizontal time grid 25 mm = 1 s). For the same 20 patients, data derived using their personalized and generalized models was also charted similarly. Figure 2 depicts a sample chart.

These charts were assigned I.D.s referred to as Chart ID, random numbers from 1 through 90. The association between the source of the data, whether actual or derived, and the I.D. numbers assigned were maintained. Cardiologists were presented with 12-lead ECG waveforms from the actual data, as well as the data derived from a subset of leads using the L2V2V6 → S15 models while they were blinded to the source of the waveforms. They were requested to provide interpretation for each of the presented charts. The interpretations were then compared between the actual and derived ECG waveforms to determine the level of agreement. Due to potential intra-observer variations, all sets of charts with any mismatches in the interpretation were simultaneously charted to analyze whether the differences are evident from the waveforms. The qualitative blinded assessment results are presented as quantitative measures of mismatches by a direct comparison of the number of mismatches that were counted for each of the four interpretation types. Namely, rhythm, conduction blocks, Anatomical findings (chamber enlargement, ischemia and associated region, and MI and associated region or time of occurrence), ST-T abnormalities, and benign findings. The total number of differences in the interpretation, including missing or additional interpretations, are counted as errors. The total number of errors is then compared between the Personalized Model (PM)-ECG and Generalized model (G.M.)-ECG. The charts where discrepancies were found and resolved are included in the Supplementary Information.

## 4. Results

### 4.1. Quantitative Assessments

**Figure 3:**
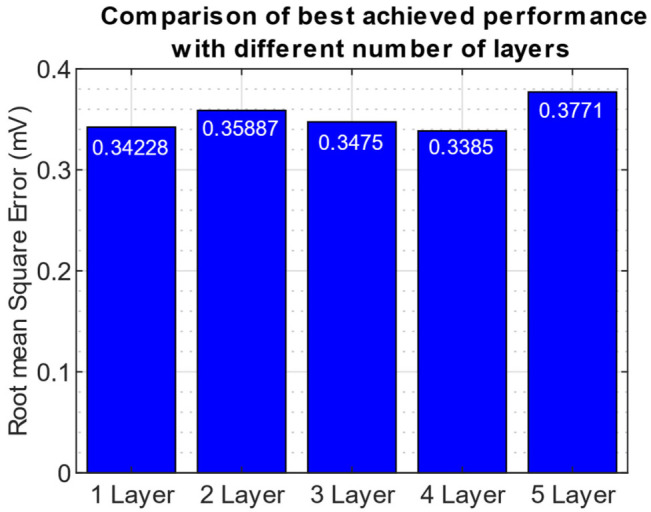
Final Test Set RMSE values for the optimal models found using BOpt.

We trained 250 neural networks as part of the BOpt experiments: 50 networks as part of each evaluation for 1-, 2-, 3-, 4-, and 5-Layer networks. The final test set RMSE values for the networks are shown in Figure 3.

We observed that the 4-layer network had the lowest test set RMSE value (0.3385 mV). The difference between the best and worst RMSE is ~40 µV. The hyperparameters associated with the optimal model are in Table 4.

**Figure 4:**
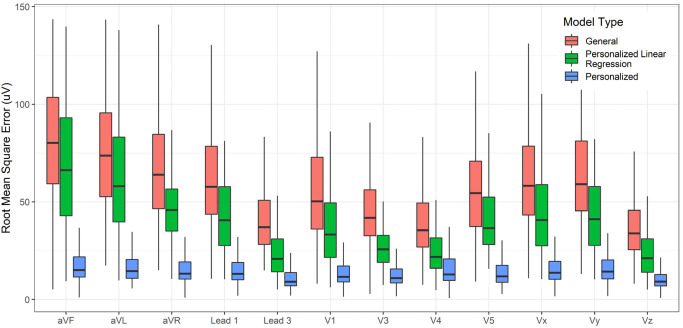
Compare the RMSE of each derived channel between a GM-ECG, personalized linear regression, and PM-ECG for Lead II, V2, and V6 to all other leads transformation model.

**Figure 5:**
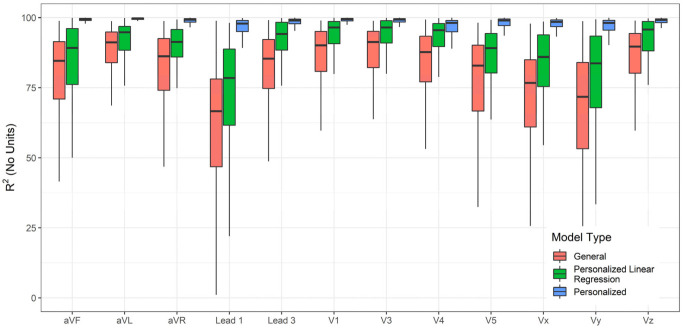
Compare the R^2^ of each derived channel between a GM-ECG, personalized linear regression, and PM-ECG for Lead II, V2, and V6 to all other leads transformation model.

**Figure 6:**
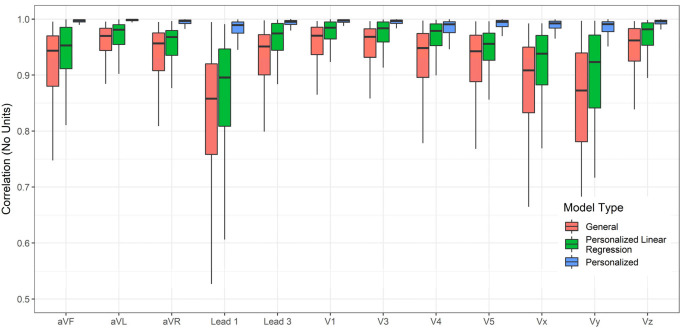
Compare the Pearson Correlation Coefficient of each derived channel between a GM-ECG, personalized linear regression, and PM-ECG for Lead II, V2, and V6 to all other leads transformation model.

The accuracy of lead transformations to Frank XYZ could be compared to several results reported in the literature (Figures S12–S14 in the Supplementary Material). However, the accuracy of conversion to all leads other than the inputs (Lead II, V2, and V6) is a novel exploration in this work. In addition to the personalized and general models, linear regression transformations for personalized data were also determined, and their performance was charted for comparison. The linear transformations were computed using Q.R. factorization [48]. Figure 4, Figure 5 and Figure 6 compare the RMSE, R^2^, and Pearson Correlation Coefficient values for all the derived leads of ECG between the generalized model, the personalized model, and linear regression applied to the personalized models. The boxplots present the median values, lower and upper quartiles, and the minimum and maximum values. The Supplementary Information provides the mean and standard deviation of RMSE, R^2^, and ρ for the general, personalized linear regression and personalized models.

### 4.2. Qualitative Assessments

As described in the methods section, since the ECG interpretations were not categorical and entered in a free-form text by the cardiologists, the interpretations were grouped by diagnostic criterion. Table 5 lists the identified error counts and the subsequent correction of errors due to intra-observer variance in parenthesis. The simultaneous charts of the ECG waveforms from actual, PM-ECG, and GM-ECG are presented in the Supplementary Information to reveal the rationale for the corrections made.

## 5. Discussion

The results obtained through this study show that the personalized models result in a more accurate derivation of the 12-lead ECG waveforms for all patients in terms of three quantifiable measures of error, namely, RMSE, R^2^, and pearson correlation coefficient. The methodology of applying transfer learning to obtain personalized models from the generalized models shows promising results in terms of quantitative accuracy of derivations. 

For the problem of designing an optimal algorithm for the derivation of all leads from a subset of leads, two broadly defined approaches could be identified. Namely, a hybrid approach, and an end-to-end approach. In the hybrid approach, we would use a priori knowledge regarding linear associations between the leads, which are established through Einthoven’s equations and use a function approximation method to derive the remaining leads. In the end-to-end approach, a single function approximation method may be used to find the relationship between a chosen set of leads and all other leads. The end-to-end approach, which is followed in this paper, selects inputs that resulted in the high-performance transformations in the literature in terms of low errors, Lead II, V2, and V6 [49]. Under the assumption that these leads form an orthogonal basis that could then be used to approximate a function to derive all other leads, LSTM models were trained to perform this task. 

Following the hybrid approach could lead to lowered power consumption overall due to lower complexity of the function approximation method, while potentially retaining or lowering the power consumption levels on the electronics that are used to acquire the signal. The end-to-end approach, on the other hand, leads to more complex computation, which could lead to greater power consumption overall. An objective comparison of these two methods is not attempted as part of this work. Such a comparison would have to account for several factors such as hardware design, choice of signal compression methods or their effectiveness, dependent on the choice of leads, implementation of the software libraries that implement the algorithms for transformations and the underlying computational hardware that supports the application, to name a few.

The most harm that could result from errors is the misinterpretation of the ECG waveforms, leading to a misdiagnosis or loss of time due to suspicion of pathologies that are not present. These can lead to delays in the appropriate treatment and deterioration of the quality of life for the patient. Therefore, a pilot assessment of the reproducibility of ECG interpretations was conducted in this study. We found that the level of agreement of the interpretations from ECG derived using personalized models and the actual ECGs was significantly higher than with generalized models. There were findings with the GM-ECG that were misleading compared to the actual ECGs and could have led to the loss of time and were potentially harmful to the patient in an actual clinical setting. Based on the findings in this study, personalized models should be the preferred approach. A more extensive study comparing ECG interpretations from a larger sample of patients with different pathologies and more board-certified cardiologists is warranted to validate these results further. The difference in diagnostic yield in terms of detail and specificity of diagnoses from a 12-lead ECG between a GM-ECG and PM-ECG was exposed in this study. The use of more complex deep learning models without accounting for a physiological difference from patient to patient could potentially propagate errors, leading to misdiagnosis. 

On balance, there are some known limitations to the application of blinded assessment as a qualitative comparison method for the level of agreement between ECG waveforms. Namely, intra-observer variability of ECG interpretations is expected because the cardiologists are, in this case, interpreting the ECG alone without patient history information. Knowledge of patient history has been shown to modulate cardiologists’ attention to specific patterns in the ECG [50]. In clinical practice, ECG interpretation alone is never used to formulate a plan for the treatment of patients, so the emphasis on diagnosis from interpretation is not representative of the standard practice. Furthermore, systematic reviews have reported that cardiologists’ aggregate accuracy of ECG interpretation is 74.9% (95% CI, 63.2–86.7%) [51]. In this study, the simultaneous charting of the ECGs from the three sources is used to rule out any differences in interpretation that are not discernible from the waveforms.

For all machine learning and deep learning approaches, the data set utilized to develop the model must be truly representative of the intended patient population. Therefore, the larger and more representative the data set used for the generalized model, the more likely it will generalize well to all patients and be more accurate. Moreover, in this study we have explored LSTM models exclusively due to the best performance reported thus far for the ECG lead transformation problem. It is possible to use more innovative deep learning architectures to discover a generalized model architecture that could provide diagnostically equivalent ECGs compared to actual ECGs, but this remains an active area of research that must validate clinical diagnostic equivalence through qualitative assessments and not just quantitative measures of error. 

However, the observations from this study suggest that the desired approach should be personalized models and not generalized models. ECGs identical to the actual ECG waveforms could be derived using the data set utilized in this study and the trained personalized models. Whether a larger data set would improve the generalized model accuracy, which would translate to a more accurate personalized model, requires further empirical evaluation. However, clinical equivalence was achievable with this data set as seen by the level of agreement of the ECG interpretations between actual and PM-ECGs.

The validity of the transformations for the same patient before and after a major cardiovascular event needs to be evaluated in a longitudinal study. Such a study would help evaluate the hypothesis that the neural network has learned the nonlinear transfer function reflective of the subject’s anatomy rather than overfitting to the data obtained at that moment in time. The nature of data available for this research does not allow that evaluation.

Finally, the proposed methodology of personalized transformations has practical limitations that must be addressed from a cost and labor perspective for healthcare providers. A personalized model for each patient will require a controlled clinical measurement of 15-lead ECG, placing all 16 electrodes to obtain the data needed to train a personalized model for each patient. Since there is added cost beyond a routine clinical indication, such a personalized approach would require a risk vs. benefit analysis to determine if the direct and indirect costs for such a procedure would be justified. To reach a stage of adoption, there would also need to be evidence to support an increased diagnostic yield using this method. 

## 6. Conclusions

An LSTM neural network was explored as a transformation method to transform a subset of ECG leads into all the 12-leads. The subset of leads chosen for this study were leads II, V2, and V6. The deep learning model trained on a large corpus of data was fine-tuned with patient-specific ECGs to yield personalized lead transformation models. These personalized transformations were evaluated to determine the extent of performance gain that can be achieved in terms of accuracy of the transformations compared to ground truth (actual measurement of biopotentials). The personalized models showed RMSE values lower than the generalized models and pearson correlation coefficient, and R^2^ values higher than the generalized models, establishing that personalized models should be preferred over generalized models from an accuracy of transformation perspective. 

Blinded assessment of the diagnostic yield of such models was explored to determine the level of agreement between the diagnosis from the actual ECG waveforms and those derived from generalized models and personalized models. The PM-ECGs were found to be diagnostically equivalent to the actual ECGs.

## Figures and Tables

**Table 3:** BOpt search space for each of the hyperparameters that were optimized.

Hyperparameter	Bounds for Optimization	Sampling Transformation
**Number of Hidden Units**	[10, 50]	Linear, Uniform
**Minibatch Size**	[16, 32]	Linear, Uniform
**Learning Rate Schedule**	‘none’ or ‘piecewise’ (reduced by a factor of 0.1 every 10 epochs)	Linear, Uniform
**Learning rate**	[1 × 10^−3^, 1 × 10^−1^]	Log-scaled, Uniform
$\beta_{1}$ **(Momentum coefficient)**	[0.9, 0.999]	Log-scaled, Uniform
$\beta_{2}$ **is RMS prop coefficient**	[0.9, 1]	Log-scaled, Uniform

**Table 4:** List of Optimal hyperparameters found through BOpt.

Number of Hidden Units	Layer 1—27 Layer 2—22 Layer 3—23 Layer 4—46
Minibatch size	27
Learning rate Schedule	None—No change to the learning rate
$\mathrm{Gradient}\;\mathrm{Decay}\;\mathrm{Factor}\;(\beta_{1}$)	0.90034
$\mathrm{Squared}\;\mathrm{Gradient}\;\mathrm{Decay}\;\mathrm{Factor}\;(\beta_{2}$)	0.9175
Learning Rate	0.028805

**Table 5:** Error Rates for ECG interpretation compared to actual ECG with correction for intra-observer errors.

Diagnostic Criterion	Actual	PM-ECG	GM-ECG	PM-ECG	GM-ECG
				(Errors in Observations (Errors after Correcting for Intra-Observer Errors))	
Rhythm	Sinus rhythm (*n* = 18)	Sinus rhythm (*n* = 18)	Sinus rhythm (*n* = 18)	2(0)	0
	Atrial fibrillation with rapid ventricular rate (*n* = 1)	Atrial fibrillation with rapid ventricular rate (*n* = 1)	Atrial fibrillation with rapid ventricular rate (*n* = 1)		
	Sinus tachycardia (*n* = 1)	Sinus tachycardia (*n* = 1)	Sinus tachycardia (*n* = 1)		
	PVC (*n* = 2)	PVC (*n* = 2)	PVC (*n* = 2)		
Conduction blocks	Left bundle branch block or LBBB (*n* = 3)	Left bundle branch block or LBBB (*n* = 3)	Left bundle branch block or LBBB (*n* = 3)	0(0)	0
	Left anterior fascicular block (*n* = 1)	Left anterior fascicular block (*n* = 1)	Left anterior fascicular block (*n* = 1)		
Anatomical findings	Left ventricular hypertrophy (*n* = 3)	Left ventricular hypertrophy (*n* = 3)	Left ventricular hypertrophy (*n* = 4, 1 error)	0(0)	1
ST-T wave findings (ischemia)	ischemia (*n* = 4)	ischemia (*n* = 4)	ischemia (*n* = 2, 2 errors)	3(0)	8
	Tall T wave (*n* = 1)	Tall T wave (*n* = 1)	Tall T wave (*n* = 0, 1 error)		
	ST depression (*n* = 1)	ST depression (*n* = 1)	ST depression (*n* = 1)		
	T wave inversion (*n* = 2)	T wave inversion (*n* = 3)	T wave inversion (*n* = 1, 2 errors)		
	T wave abnormality (*n* = 3)	T wave abnormality (*n* = 2)	T wave abnormality (*n* = 3, 2 findings don’t match with actual ECG, total errors = 3)		
MI region and time of occurrence	recent anterior MI (*n* = 1)	recent anterior MI (*n* = 1)	recent anterior MI (*n* = 1, 1 error)	1(0)	4
	recent inferior MI (*n* = 1)	recent inferior MI (*n* = 1)	recent inferior MI (*n* = 0, 1 error)		
	old inferior MI (*n* = 1)	old inferior MI (*n* = 2, 1 error)	old inferior MI (*n* = 3, 2 errors)		
Miscellaneous or benign findings	left axis (*n* = 1)	left axis (*n* = 1) prominent U waves (*n* = 0) left atrial enlargement (*n* = 0)	left axis (*n* = 0) early repolarization (*n* = 0)	3(0)	2(0)
Total errors				9 (0)	15 (13)

## Data Availability

Data used in this study is publicly available from the PhysioNet Database.

## Supplementary Materials

The following information can be downloaded at https://www.mdpi.com/article/10.3390/s23031389/s1, Figure S1: Diagnosis from actual ECG—Normal sinus rhythm, T wave abnormality consider ischemia, PVC.; Diagnosis from generalized model—Sinus rhythm, consider anterior ST elevation MI, LVH, PVC The red markers indicate the regions in the GM- ECG that deviates from the actual and PM-ECG.; Figure S2: Actual diagnosis -Normal sinus rhythm, LVH with secondary repolarization changes; Personalized model diagnosis—Normal sinus rhythm, LVH with secondary repolarization changes, old inferior MI; Generalized model diagnosis—Normal sinus rhythm, LVH with secondary repolarization changes, probably old inferior MI. The red markers indicate the regions in the GM- ECG that deviates from the actual and PM-ECG.; Figure S3: Actual diagnosis—Sinus rhythm, consider acute/recent anterior ST elevation MI; Personalized model diagnosis—Sinus tachycardia, Acute/recent anterior ST elevation MI; Generalized model diagnosis—Sinus rhythm, consider acute/recent anterior ST elevation MI, probable old inferior MI. The red markers indicate the regions in the GM- ECG that deviates from the actual and PM-ECG.; Figure S4: Actual diagnosis—Normal sinus rhythm, T wave inversion suggestive of ischemia; Personalized model diagnosis—Normal sinus rhythm, T wave inversion suggestive of ischemia; Generalized model diagnosis—Normal sinus rhythm, Left ventricular hypertrophy with secondary repolarization changes. The red markers indicate the regions in the GM- ECG that deviates from the actual and PM-ECG.; Figure S5: Actual diagnosis—Normal sinus rhythm, Left Ventricular Hypertrophy with secondary repolarization, probably acute/recent Inferior myocardial infarction; Personalized model diagnosis Normal sinus rhythm, Left Ventricular Hypertrophy with secondary repolarization, probably acute/recent Inferior myocardial infarction; Generalized model diagnosis—Normal sinus rhythm, non-specific T wave abnormality. The red markers indicate the regions in the GM- ECG that deviates from the actual and PM-ECG.; Figure S6: Actual diagnosis—Normal sinus rhythm, Tall T waves consider hyperkalemia; Personalized model diagnosis Normal sinus rhythm, Tall T waves consider hyperkalemia; Generalized model diagnosis—Normal sinus rhythm, non-specific T wave abnormality. The red markers indicate the regions in the GM- ECG that deviates from the actual and PM-ECG.; Figure S7: Actual diagnosis—Normal sinus rhythm, normal ECG; Personalized model diagnosis Normal sinus rhythm, prominent U waves; Generalized model diagnosis—Normal sinus rhythm, early repolarization. The red markers indicate the regions in the GM- ECG that deviates from the actual and PM-ECG.; Figure S8: Actual diagnosis—Normal sinus rhythm, old inferior MI; Personalized model diagnosis Normal sinus rhythm, old inferior MI, left atrial enlargement; Generalized model diagnosis—Normal sinus rhythm, old inferior MI. The red markers indicate the regions in the GM- ECG that deviates from the actual and PM-ECG.; Figure S9: Actual diagnosis –Sinus rhythm, low amplitude, T wave changes, possible anterior wall ischemia; Personalized model diagnosis Sinus rhythm, T inversion, possible anterior wall ischemia, low amplitude QRS complexes; Generalized model diagnosis—Sinus low amplitude QRS, possible anterior wall ischemia. The red markers indicate the regions in the GM- ECG that deviates from the actual and PM-ECG.; Figure S10: Actual diagnosis –Sinus rhythm, left axis, likely left anterior fascicular block, diffuse T wave inversion; Personalized model diagnosis Sinus rhythm, likely left anterior fascicular block, diffuse T wave inversion; Generalized model diagnosis—Sinus rhythm, likely left anterior fascicular block, diffuse T wave inversion. The red markers indicate the regions in the GM- ECG that deviates from the actual and PM-ECG.; Figure S11: Actual diagnosis –Normal sinus rhythm; Personalized model diagnosis Sinus rhythm, tall T waves; Generalized model diagnosis—Sinus rhythm. The red markers indicate the regions in the GM- ECG that deviates from the actual and PM-ECG; Figure S12: Comparison of Correlation Coefficients for deriving Frank XYZ from standard 12 lead and the proposed GM-ECG and PM-ECG models; Figure S13: Comparison of RMSE for deriving Frank XYZ from standard 12 lead and the proposed GM-ECG and PM-ECG models; Figure S14: Comparison of R2 for deriving Frank XYZ from standard 12 lead and the proposed GM-ECG and PM-ECG models; Table S1: Comparison of ECG interpretations from the three sources of ECG; Table S2: RMSE (mean ± std) for the derivation of all leads using the general and personalized Lead 2, V2, V6 to all other leads transformations; Table S3: R2 (mean ± std) for the derivation of all leads using the general and personalized Lead 2, V2, V6 to all other leads transformations; Table S4: Pearson Correlation Coefficient (mean ± std) for the derivation of all leads using the general and personalized Lead 2, V2, V6 to all other leads transformations; Pseudocode for Hyperparameter search using Bayesian Optimization.
