# Supplementary material for: Personalized LSTM Models for ECG Lead Transformations Led to Fewer Diagnostic Errors Than Generalized Models: Deriving 12-Lead ECG from Lead II, V2, and V6
Source: Sensors (Basel). 2023 Jan 26;23(3):1389. doi: 10.3390/s23031389 (PMC9920327; doi:10.3390/s23031389)
Supplement: Supplementary file 1 [file sensors-23-01389-s001.zip › sensors-2094681-supplementary.pdf]

**Diagnostic Assessment**

Table S1. lists the interpretation notes of the cardiologists for the 20 patients. Each row refers to a patient. The table includes the chart ID presented for the actual ECG, ECG derived by the personalized model specific to the patient, and the general model applicable to all patients. The mismatched interpretations are highlighted in **bold red font**. Subsequently, the mismatched ECGs are charted simultaneously to reveal the differences in the underlying waveforms that lead to the difference in interpretation.

**Table S1 Comparison of ECG interpretations from the three sources of ECG**

| Actual | Actual Chart ID | Personalized Model derived ECG (PM-ECG) | Personalized Chart ID | General Model derived ECG (GM-ECG) | General Chart ID |
|---|---|---|---|---|---|
| Normal sinus rhythm, Nonspecific T wave abnormality | 67 | Normal sinus rhythm, Nonspecific T wave abnormality | 77 | Normal sinus rhythm, Nonspecific ST abnormality | 78 |
| Normal sinus rhythm, T wave abnormality consider ischemia, PVC. | 68 | Normal sinus rhythm, T wave abnormality consider ischemia, PVC. | 73 | Sinus rhythm, **consider anterior ST elevation MI, LVH**, PVC | 46 |
| Normal sinus rhythm, normal ECG | 69 | Normal sinus rhythm, normal ECG | 36 | Normal sinus rhythm, normal ECG | 51 |
| Normal sinus rhythm, LVH with secondary repolarization changes | 84 | Normal sinus rhythm, LVH with secondary repolarization changes, **old inferior MI** | 65 | Normal sinus rhythm, LVH with secondary repolarization, **probably old Inferior myocardial infarction** | 5 |
| Sinus rhythm, consider acute/recent anterior ST elevation MI | 57 | **Sinus tachycardia**, Acute/recent anterior ST elevation MI | 13 | Sinus rhythm, consider acute/recent anterior ST elevation MI, **probable old inferior MI** | 49 |
| Normal sinus rhythm, T wave inversion suggestive of ischemia | 23 | Normal sinus rhythm, T wave inversion suggestive of ischemia | 16 | Normal sinus rhythm, **LVH with secondary repolarization changes** | 76 |
| Normal sinus rhythm, LVH with secondary repolarization, probably acute/recent Inferior myocardial infarction | 4 | Normal sinus rhythm, LVH with secondary repolarization, probably acute/recent Inferior myocardial infarction | 81 | Normal sinus rhythm, **Nonspecific ST-T wave abnormality** | 64 |
| Normal sinus rhythm, Tall T waves consider hyperkalemia | 18 | Normal sinus rhythm, Tall T waves consider hyperkalemia | 88 | Normal sinus rhythm, **nonspecific T wave abnormality** | 14 |
| Normal sinus rhythm, normal ECG | 8 | Normal sinus rhythm, **prominent U waves** | 31 | Normal sinus rhythm, **Early repolarization pattern** | 3 |
| Normal sinus rhythm, old inferior MI | 20 | Normal sinus rhythm, old inferior MI, **left atrial enlargement** | 7 | Normal sinus rhythm, old inferior MI | 66 |
| sinus rhythm, frequent PVCs of same morphology (monomorphic) | 37 | sinus rhythm, frequent PVCs of same morphology(monomorphic) | 32 | sinus, frequent monomorphic PVCs | 59 |
| sinus rhythm, low amplitude, T wave changes, possible anterior wall ischemia | 58 | sinus rhythm, **T inversion** possible anterior wall ischemia, low amplitude QRS complexes | 50 | sinus, low amplitude QRS, possible anterior wall ischemia | 54 |
| sinus rhythm, LVH with repolarization changes | 29 | sinus rhythm, Left ventricular hypertrophy with repolarization changes. | 90 | sinus rhythm, Left ventricular hypertrophy with repolarization changes. | 12 |
| atrial fibrillation with rapid ventricular rate, LBBB | 33 | atrial fibrillation with rapid ventricular rate, LBBB | 86 | Atrial fibrillation with rapid ventricular rate, LBBB | 74 |
| sinus with LBBB | 45 | sinus rhythm, LBBB | 85 | sinus rhythm, LBBB | 40 |

| Actual | Actual Chart ID | Personalized Model derived ECG (PM-ECG) | Personalized Chart ID | General Model derived ECG (GM-ECG) | General Chart ID |
|---|---|---|---|---|---|
| sinus tachycardia, LBBB | 53 | sinus tachycardia, left bundle branch block | 17 | sinus tachycardia, LBBB | 63 |
| sinus rhythm, **left axis**, likely left anterior fascicular block, diffuse T wave inversion | 52 | sinus rhythm, left anterior fascicular block, diffuse T wave inversions | 72 | sinus rhythm, left anterior fascicular block, diffuse T inversion in precordial leads (if female sex- meets LVH criteria) | 21 |
| sinus rhythm, ST depression lateral leads possible ischemia | 30 | sinus, ST depression lateral leads, possible lateral wall ischemia | 62 | sinus rhythm, ST depression lateral leads, possible lateral wall ischemia | 61 |
| sinus rhythm | 25 | normal sinus rhythm | 89 | normal sinus rhythm | 55 |
| normal sinus rhythm | 70 | Sinus rhythm, **Tall T waves** | 6 | Sinus rhythm | 48 |

In the set of chart IDs 68, 73, and 46, the actual and PM-ECGs matched well, but the GM-ECG did not match. Figure S1 shows the simultaneously charted data. Greater than 2mm upsloping ST segment elevation seen in leads V1through V4 which represent the anterior wall and the injury pattern led to the additional consideration of “anterior ST elevation MI”. “LVH” was also considered based on the Cornell voltage criteria : R wave in aVL and the S wave in V3. If the sum is greater than 28 millimeters in males or greater than 20 mm in females

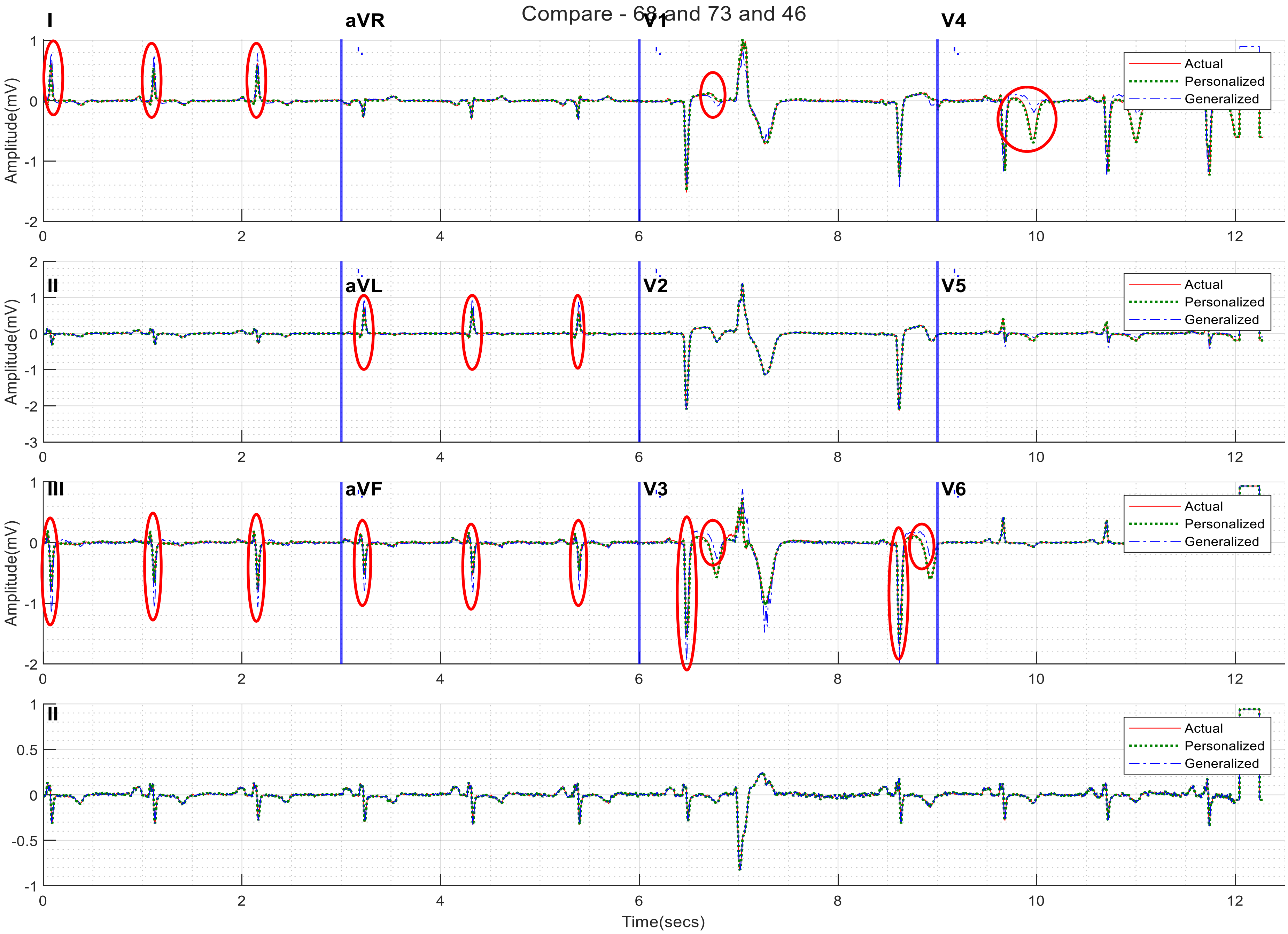


**Figure S1. Diagnosis from actual ECG - Normal sinus rhythm, T wave abnormality consider ischemia, PVC.; Diagnosis from generalized model - Sinus rhythm, consider anterior ST elevation MI, LVH, PVC The red markers indicate the regions in the GM- ECG that deviates from the actual and PM-ECG.**

In the set of chart IDs 84, 65, and 5, the actual ECG interpretation did not match PM-ECG or GM-ECG interpretations. An additional suspicion of an old inferior MI was introduced. Figure S2 shows the simultaneously charted data. The waveforms show a difference in T wave morphology between the GM-ECG and other ECGs, but no difference between the PM-ECG and actual ECGs. Old inferior MI is suspected due to Q waves in Lead III and AVF. Lead II, III and AVF are inferior leads and represent inferior wall . If seen in two contiguous leads, they satisfy criteria for an infarction . They are pathologic if they are abnormally wide (>0.2 second) or abnormally deep (>5 mm). Since the PM-ECG morphology is identical to the actual ECG, actual ECG interpretations could have also considered an old inferior MI.

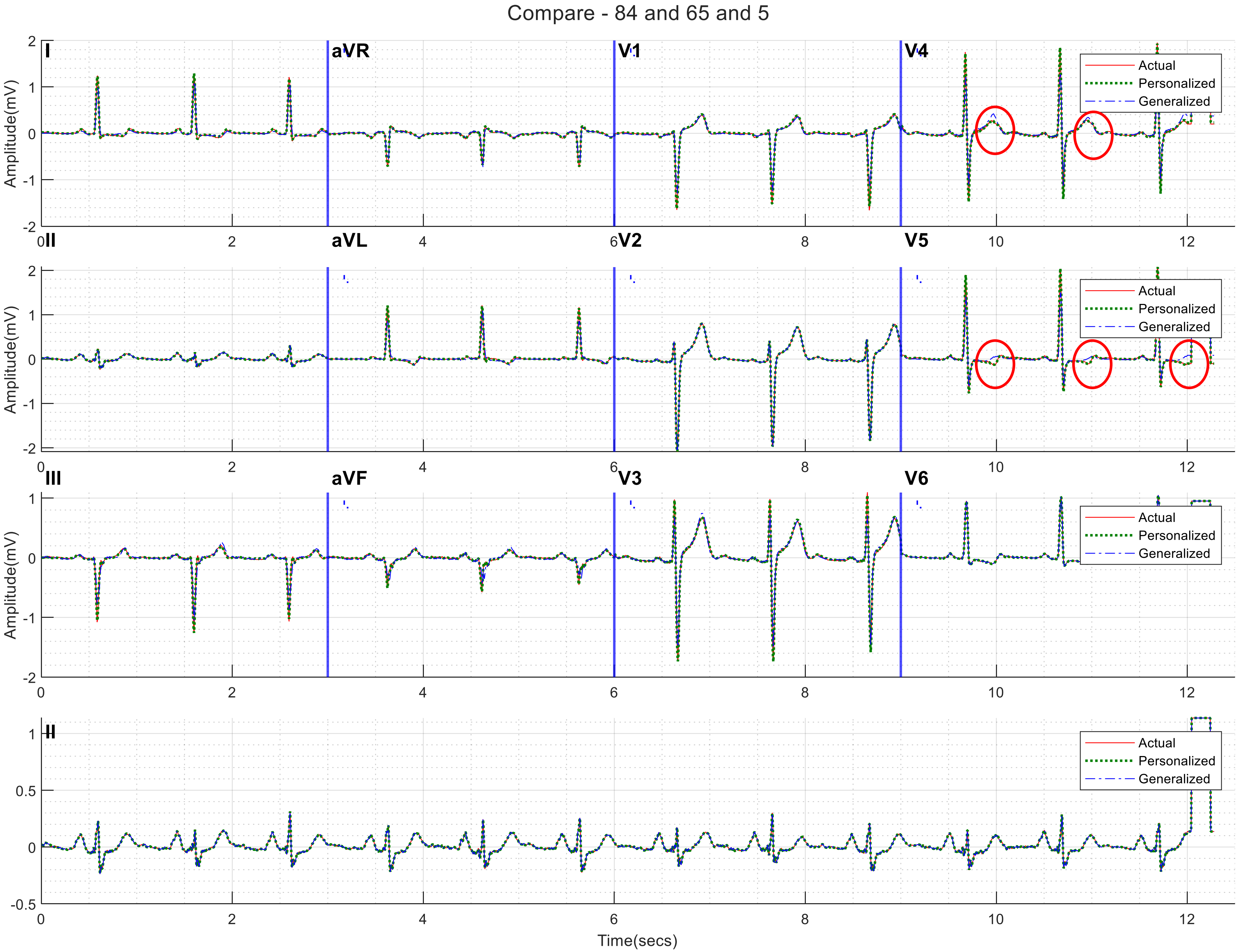


**Figure S2. Actual diagnosis -Normal sinus rhythm, LVH with secondary repolarization changes; Personalized model diagnosis - Normal sinus rhythm, LVH with secondary repolarization changes, old inferior MI; Generalized model diagnosis - Normal sinus rhythm, LVH with secondary repolarization changes, probably old inferior MI. The red markers indicate the regions in the GM- ECG that deviates from the actual and PM-ECG.**

In the chart IDs 57, 13, and 49, the actual and PM-ECGs did not match in terms of sinus rhythm. There is further suspicion of an old inferior MI for the GM-ECG. Figure S3 shows the simultaneously charted data. The waveforms show that the difference in sinus rhythm results from observer variance because the rhythm matches perfectly between all three ECGs. Old inferior MI is suspected due to Q waves in Lead III and AVF. Lead II, III and AVF are inferior leads and represent inferior wall . If seen in two contiguous leads, they satisfy criteria for an infarction . They are pathologic if they are abnormally wide (>0.2 second) or abnormally deep (>5 mm).

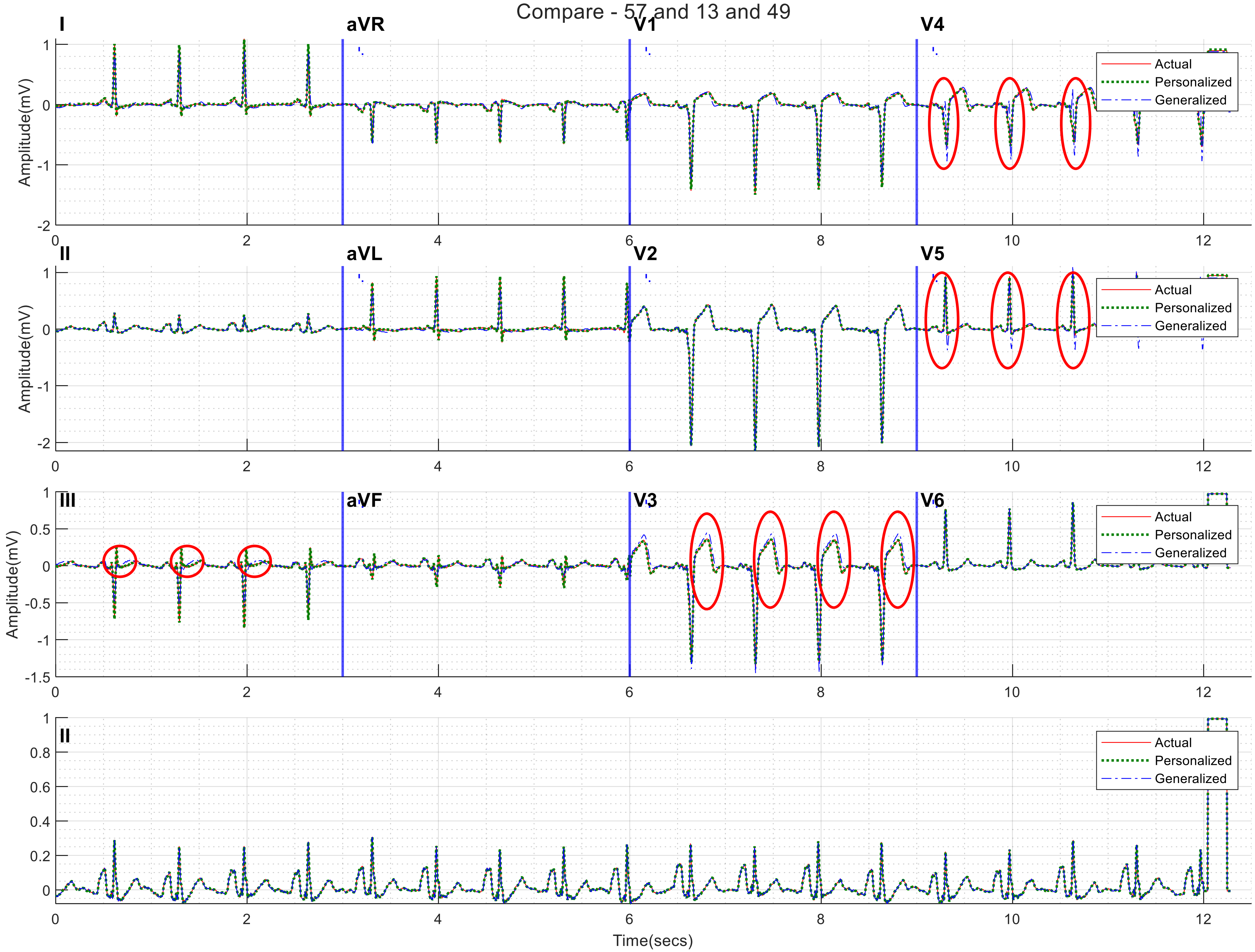


**Figure S3. Actual diagnosis - Sinus rhythm, consider acute/recent anterior ST elevation MI; Personalized model diagnosis - Sinus tachycardia, Acute/recent anterior ST elevation MI; Generalized model diagnosis - Sinus rhythm, consider acute/recent anterior ST elevation MI, probable old inferior MI. The red markers indicate the regions in the GM- ECG that deviates from the actual and PM-ECG.**

In the set of chart IDs 23, 16, and 76, the actual and PM-ECGs interpretations match entirely. Interpretation for the GM-ECG introduces new possible diagnoses of Left Ventricular Hypertrophy (LVH) with secondary repolarization changes. Figure S4 shows the simultaneously charted data. The T wave polarity and extent of inversion are different, as identified in the figure. Specifically, LVH was suspected based on the Sokolov-Lyon criteria: S wave depth in V1 + tallest R wave height in V5-V6 > 35 mm (3.5mV). Here v1 + v6 is greater than 35. Additionally, it can be seen that the T wave inversions in v4,v5 v6  the setting of LVH which is a very typical pattern of repolarization changes in LVH.

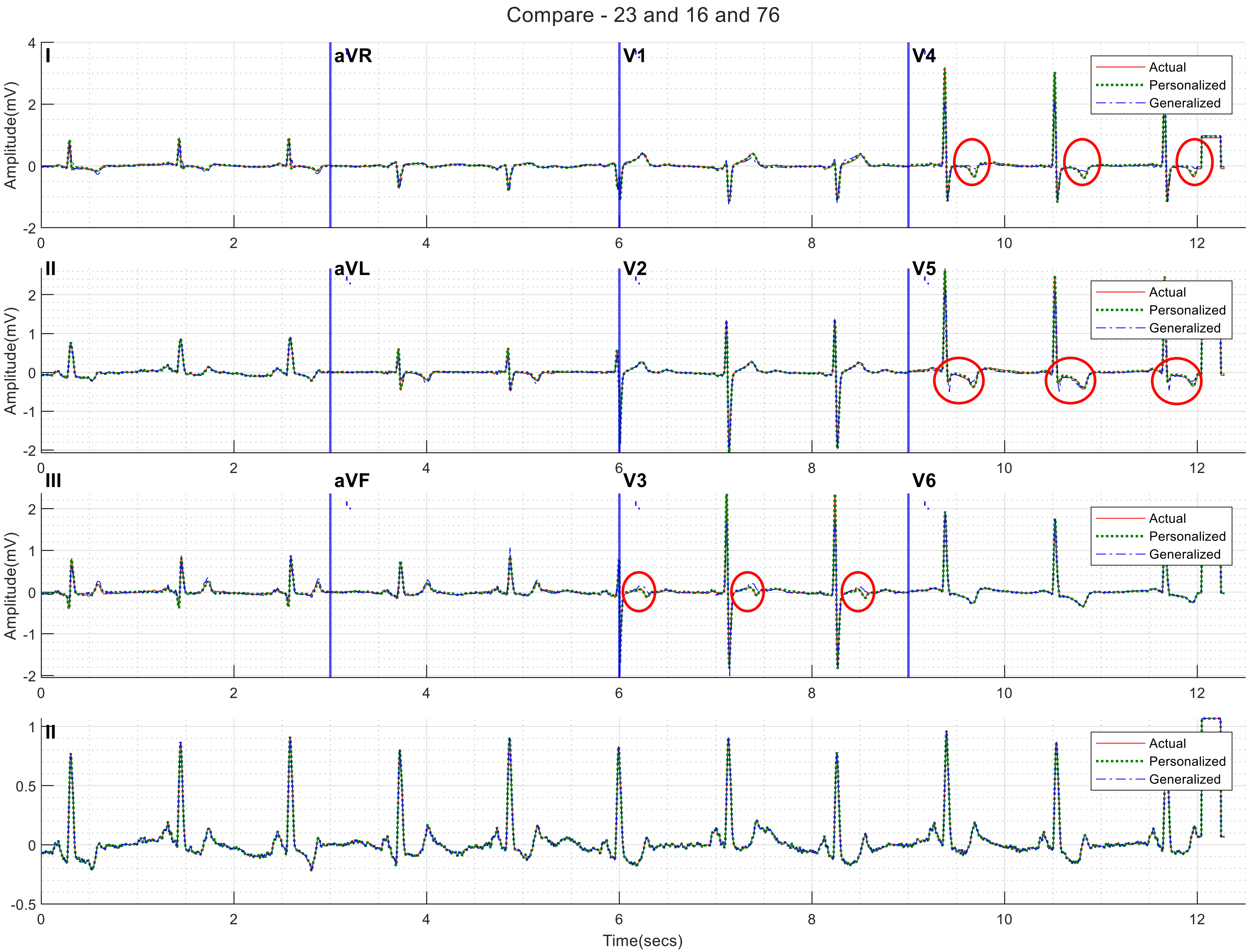


**Figure S4. Actual diagnosis – Normal sinus rhythm, T wave inversion suggestive of ischemia; Personalized model diagnosis - Normal sinus rhythm, T wave inversion suggestive of ischemia; Generalized model diagnosis – Normal sinus rhythm, Left ventricular hypertrophy with secondary repolarization changes. The red markers indicate the regions in the GM- ECG that deviates from the actual and PM-ECG.**

In the set of chart IDs 4, 81, and 64, the actual and PM-ECGs interpretations match entirely. Interpretation for the GM-ECG introduces ambiguity with nonspecific abnormalities in the ST-T wave. Figure S5 shows the simultaneously charted data. The T wave polarity is different in the GM-ECGs. Specifically, in the GM-ECGs, There are ST depressions in V5-V6 which are less than <2mm in the chest leads. If >2mm or horizontal , or reciprocal changes , then they would be specific – otherwise they are non specific.

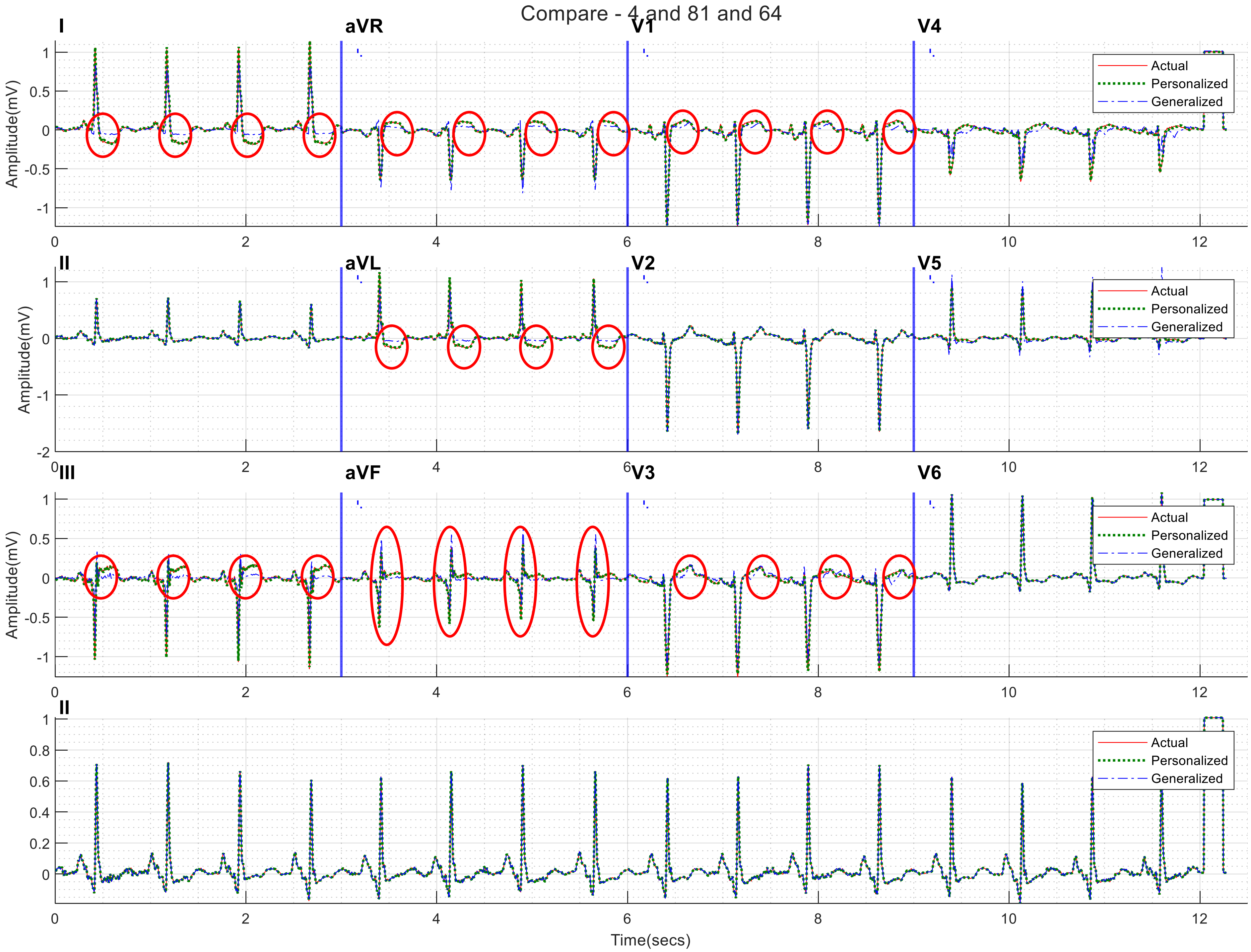


**Figure S5. Actual diagnosis – Normal sinus rhythm, Left Ventricular Hypertrophy with secondary repolarization, probably acute/recent Inferior myocardial infarction; Personalized model diagnosis Normal sinus rhythm, Left Ventricular Hypertrophy with secondary repolarization, probably acute/recent Inferior myocardial infarction; Generalized model diagnosis – Normal sinus rhythm, non-specific T wave abnormality. The red markers indicate the regions in the GM- ECG that deviates from the actual and PM-ECG.**

In the set of chart IDs 18, 88, and 14, the actual and PM-ECGs interpretations match entirely. Interpretation for the GM-ECG introduces new findings of nonspecific T wave abnormality. Figure S6 shows the simultaneously charted data. The T wave polarity, amplitude, and instances of inversion in the lead I and aVL are different, as identified in the figure. These changes led to the difference in findings. A normal T wave is upright in all leads except aVR and V1 Amplitude < 5mm in limb leads, < 10mm in precordial leads (10mm males, 8mm females). With additional information on the clinical scenario, this ECG may represent different possible diagnoses. If not other criteria are matched this can be non-specific which was the interpretation made (but this could even indicate LVH with repol if criteria are met).

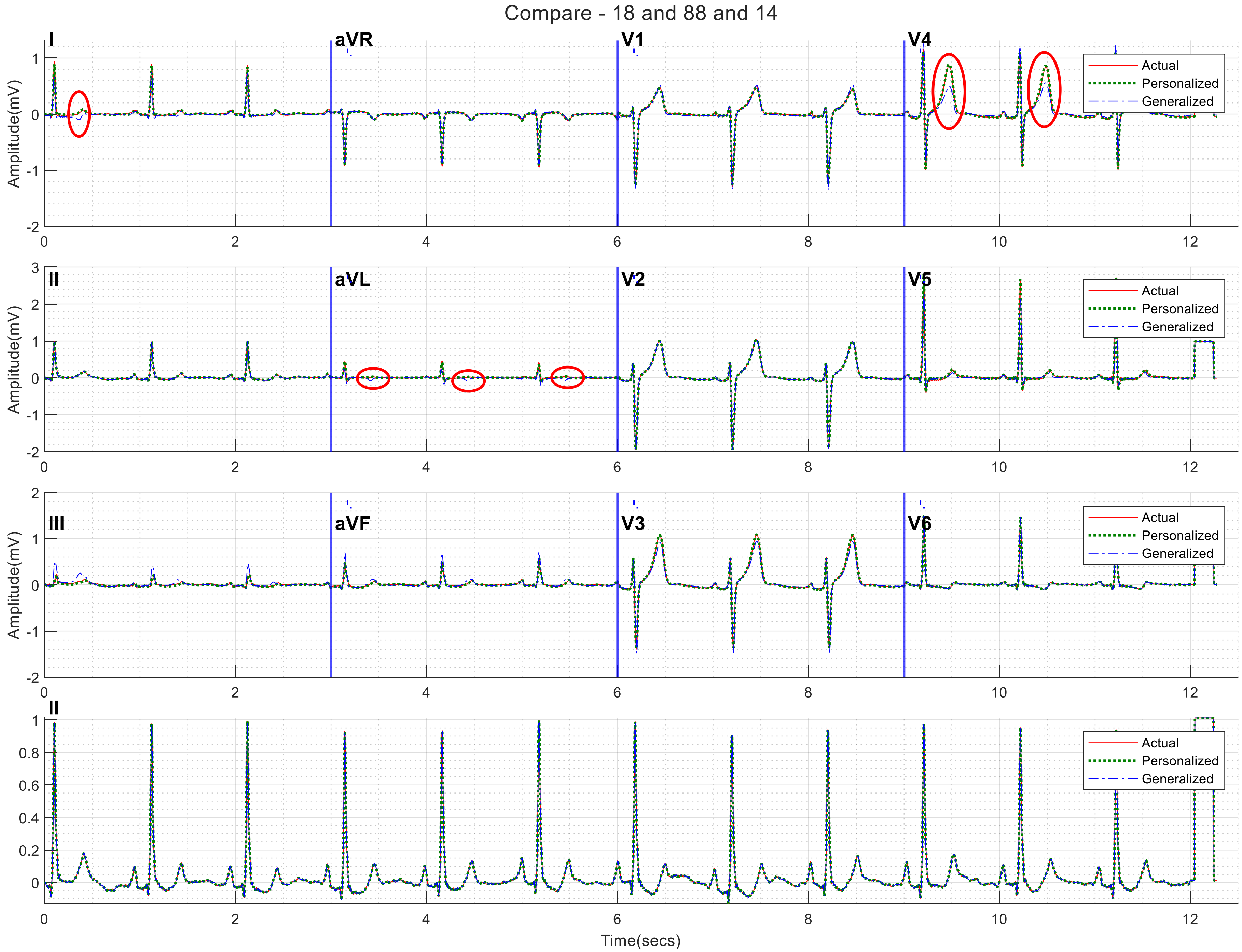


**Figure S6. Actual diagnosis – Normal sinus rhythm, Tall T waves consider hyperkalemia; Personalized model diagnosis Normal sinus rhythm, Tall T waves consider hyperkalemia; Generalized model diagnosis – Normal sinus rhythm, non-specific T wave abnormality. The red markers indicate the regions in the GM- ECG that deviates from the actual and PM-ECG.**

In the set of chart IDs 8, 31, and 3, the actual and PM-ECG interpretations differ with the additional identification of prominent U waves in the PM-ECG. In the GM-ECG, there is suspicion of early repolarization. Figure S7 shows the simultaneously charted data. From the simultaneously charted ECGs, U waves are observed in both the actual ECGs and the PM-ECG. In the GM-ECGs, LVH Sokolov-Lyon criteria was met: S wave depth in V1 + tallest R wave height in V5-V6 > 35 mm (3.5 mV). Here v1 + v6 is greater than 35 mm (3.5 mV). Upsloping ST segment in v3 v4 in setting of LVH indicates a pattern of early repolarization. Additionally, widespread concave ST elevation, most prominent in the mid-to-left precordial leads (V2-4). Notching or slurring at the J point. Prominent, slightly asymmetrical T waves that are concordant with the QRS complex.

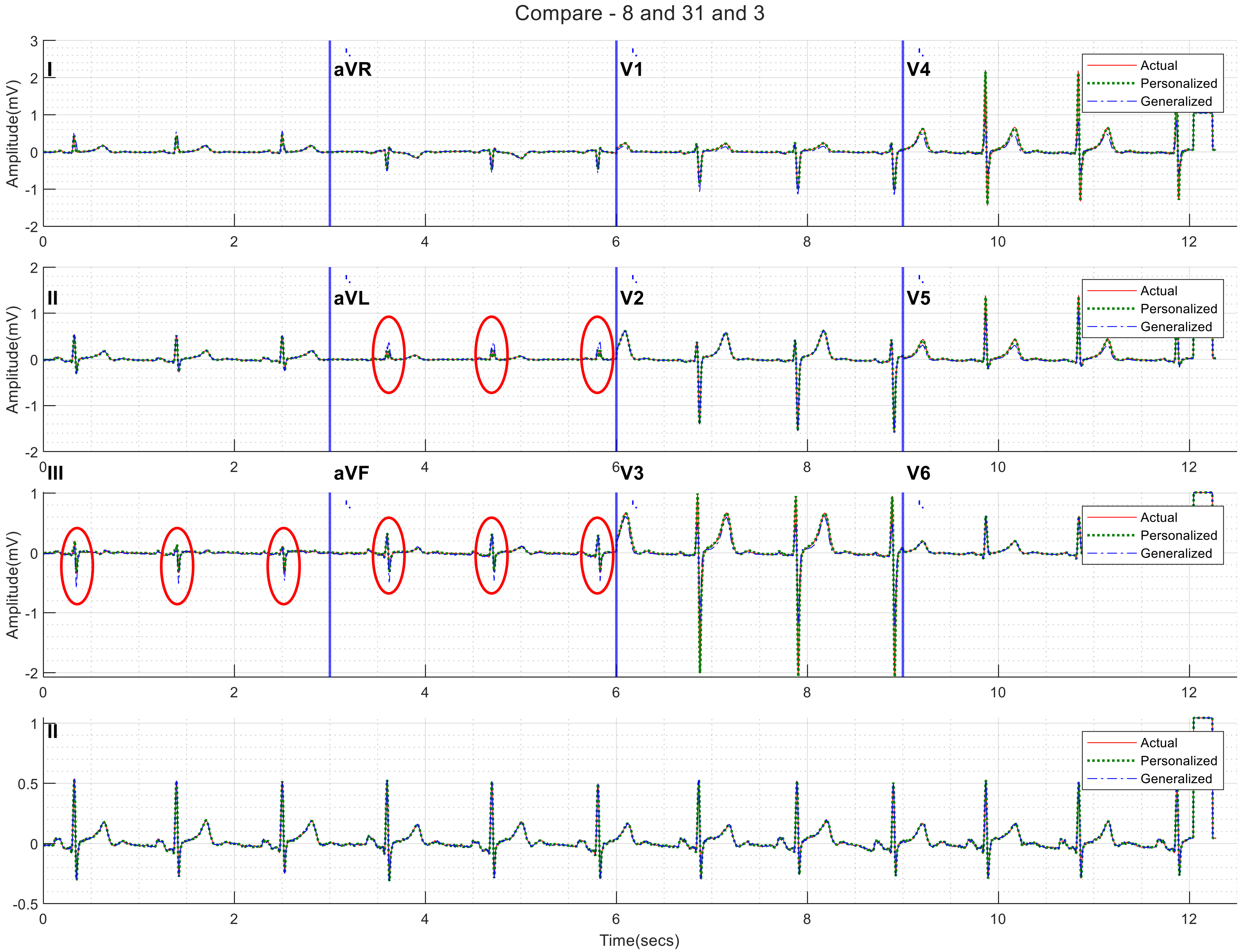


**Figure S7. Actual diagnosis – Normal sinus rhythm, normal ECG; Personalized model diagnosis Normal sinus rhythm, prominent U waves; Generalized model diagnosis – Normal sinus rhythm, early repolarization. The red markers indicate the regions in the GM- ECG that deviates from the actual and PM-ECG.**

In the set of chart IDs 20, 7, and 66, the actual and PM-ECG interpretations differ with the additional suspicion of left atrial enlargement in the PM-ECG. Figure S8 shows the simultaneously charted data. The bifid pattern of p-wave is evident in precordial lead V1 from the simultaneously charted ECGs, so this difference is attributable to observer variance. Specifically, In V1, biphasic P wave with terminal negative portion > 40 ms duration. Biphasic P wave with terminal negative portion > 1mm (0.1mV) deep. Other differences in ECG across the sources are not consistent in more than one lead, so they do not lead to a potential of misinterpretation.

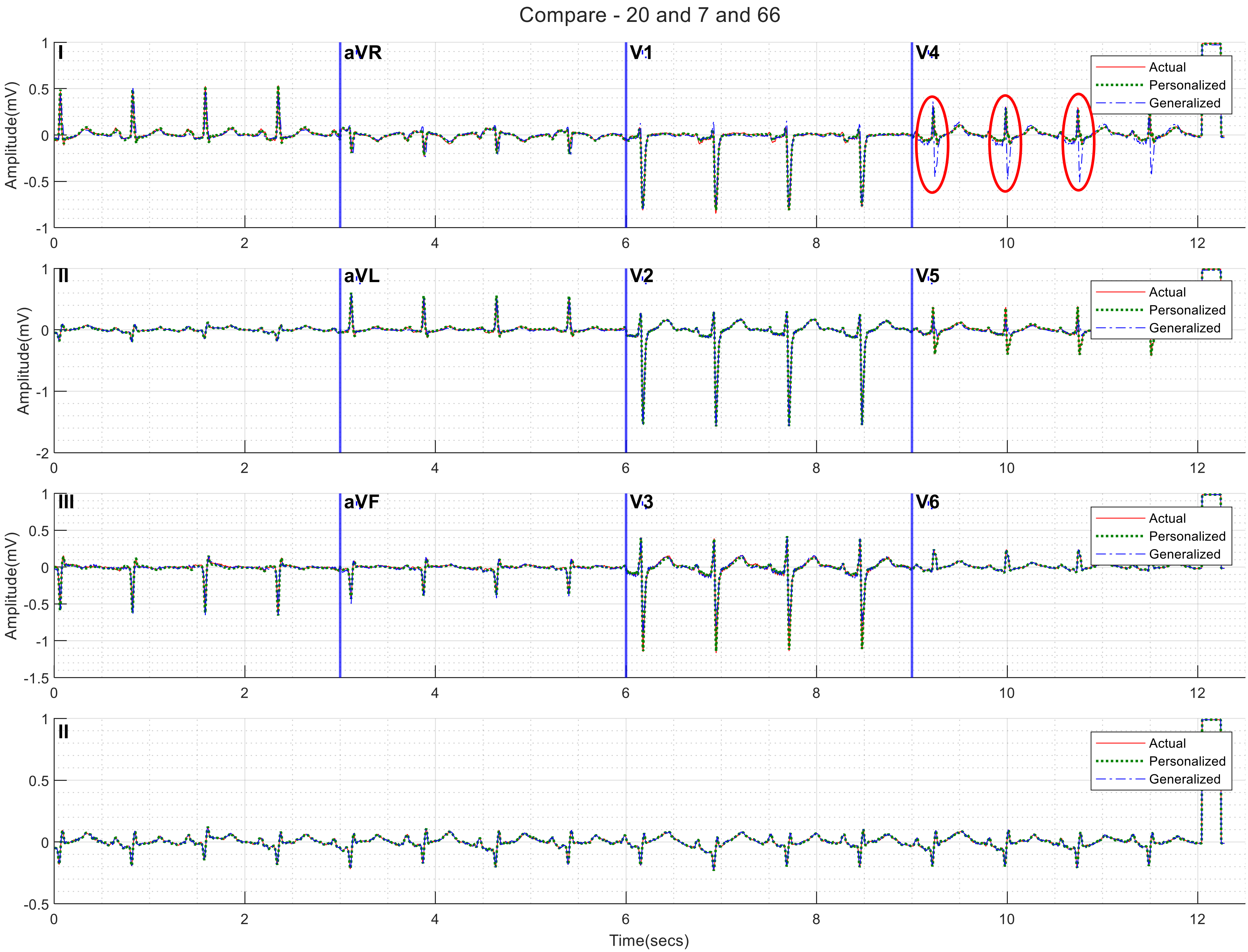


**Figure S8. Actual diagnosis – Normal sinus rhythm, old inferior MI; Personalized model diagnosis Normal sinus rhythm, old inferior MI, left atrial enlargement; Generalized model diagnosis – Normal sinus rhythm, old inferior MI. The red markers indicate the regions in the GM- ECG that deviates from the actual and PM-ECG.**

In the set of chart IDs 58, 50, and 54, the actual and PM-ECGs interpretations differ on an interpretation of the T wave morphology as “T wave changes” and “T inversion,” respectively. Figure S9 shows the simultaneously charted data. The deviations of GM-ECG T waves from PM-ECG and actual ECGs are clear in multiple leads from the simultaneously charted ECGs. However, the difference in T wave morphology between actual ECG and PM-ECG is not discernible. Therefore, this difference is attributable to observer variance.

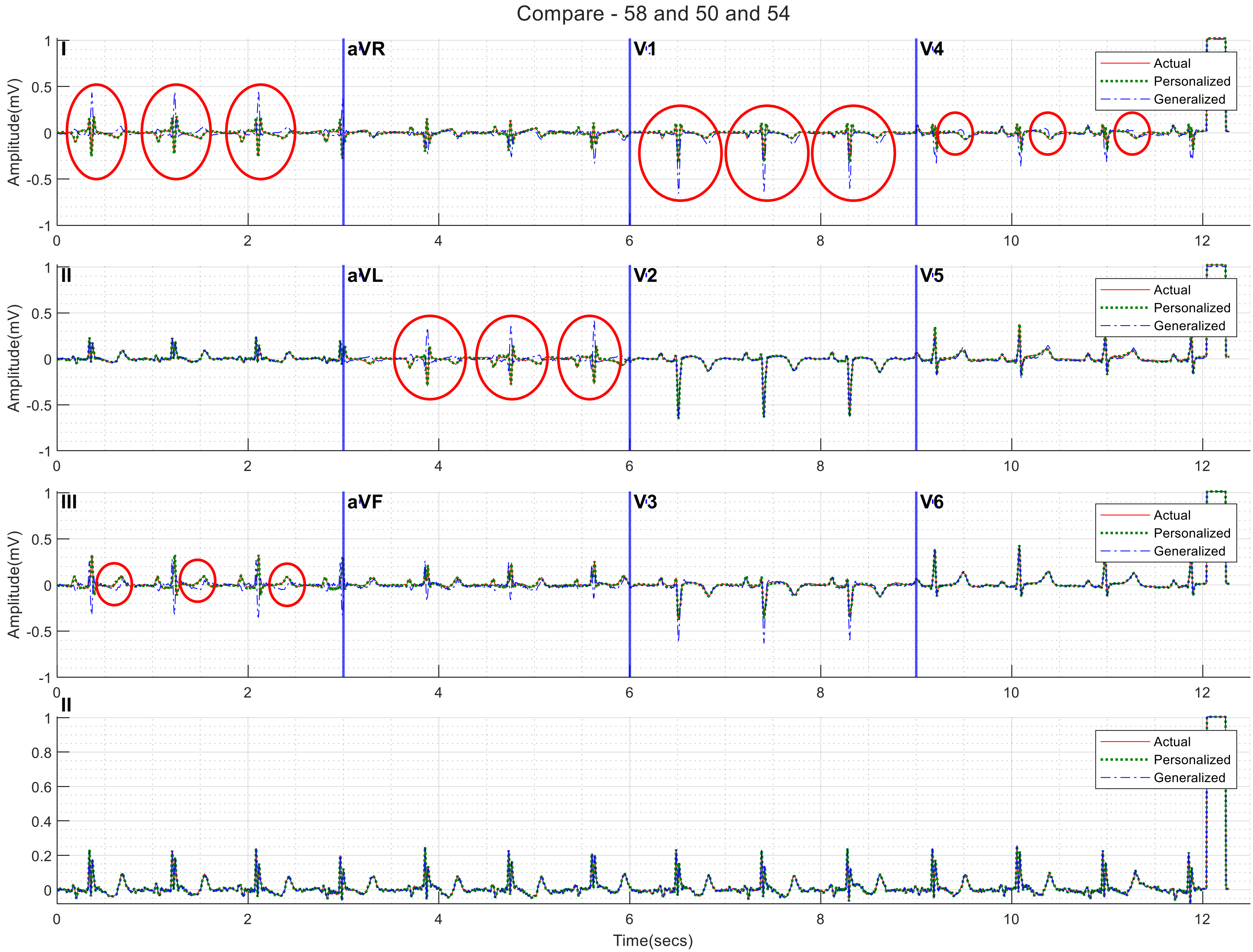


**Figure S9. Actual diagnosis –Sinus rhythm, low amplitude, T wave changes, possible anterior wall ischemia; Personalized model diagnosis Sinus rhythm, T inversion, possible anterior wall ischemia, low amplitude QRS complexes; Generalized model diagnosis – Sinus low amplitude QRS, possible anterior wall ischemia. The red markers indicate the regions in the GM- ECG that deviates from the actual and PM-ECG.**

In the set of chart IDs 52, 72, and 21, the actual and PM-ECG interpretations differ with the additional interpretation of the left axis in the actual ECG. Figure S10 shows the simultaneously charted data. Left axis deviation is observed as Leads I and aVL are positive; leads II and aVF are negative. The left axis deviation pattern is present in all ECGs from the simultaneously charted ECGs. Therefore, this difference is attributable to observer variance.

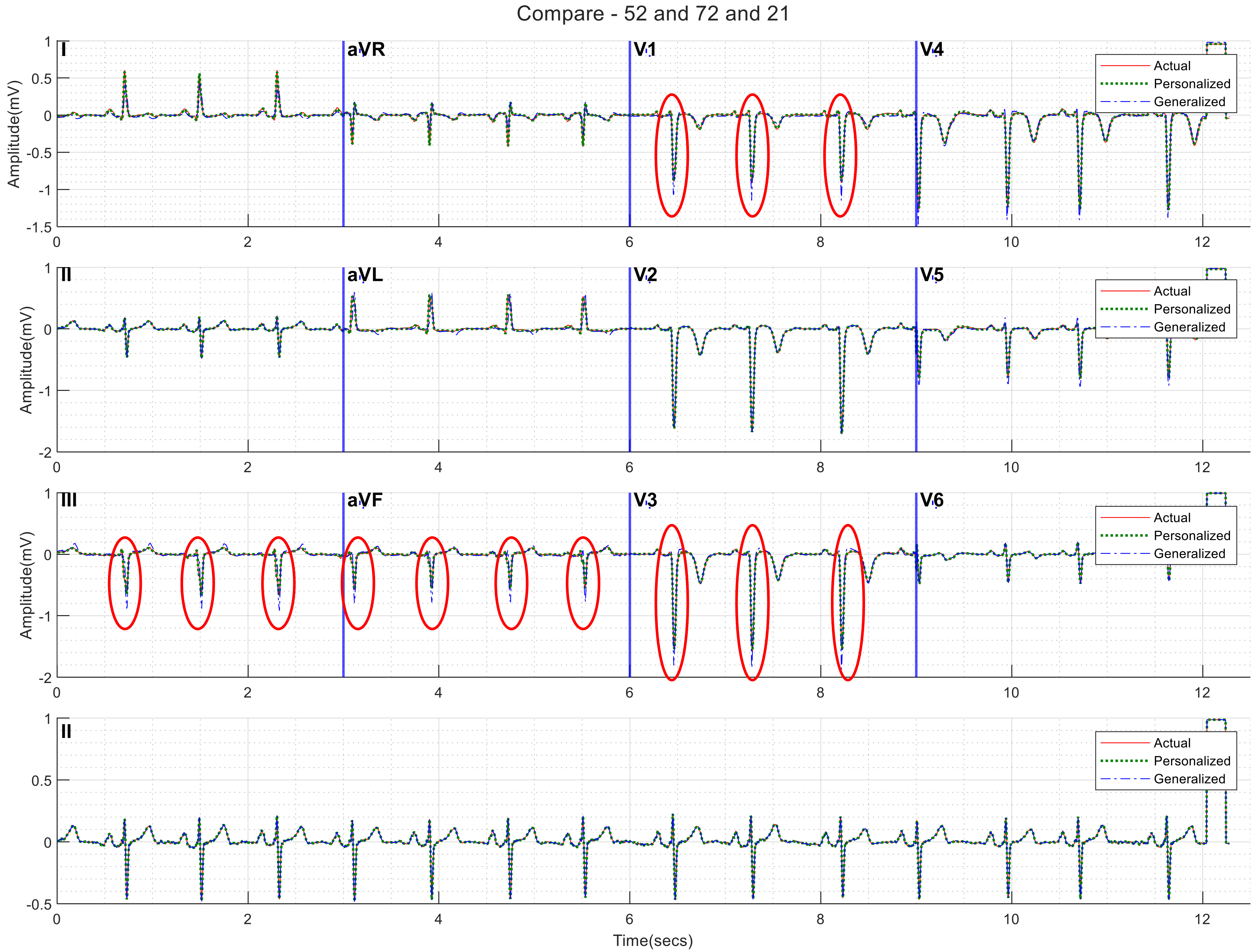


**Figure S10. Actual diagnosis –Sinus rhythm, left axis, likely left anterior fascicular block, diffuse T wave inversion; Personalized model diagnosis Sinus rhythm, likely left anterior fascicular block, diffuse T wave inversion; Generalized model diagnosis – Sinus rhythm, likely left anterior fascicular block, diffuse T wave inversion. The red markers indicate the regions in the GM- ECG that deviates from the actual and PM-ECG.**

In the chart IDs 70, 6, and 48, the actual and PM-ECG interpretations differ with the additional interpretation of tall T waves in the PM-ECGs. Figure S11 shows the simultaneously charted data. From the simultaneously charted ECGs, the T wave morphology and amplitudes seem identical between the actual ECGs and the ECGs derived from the personalized models. A normal T wave is described as upright in all leads except aVR and V1 Amplitude < 5mm in limb leads, < 10mm in precordial leads (10mm males, 8mm females). Based on this T waves are tall, but it can be further interpreted if additional clinical information is available. For example, this is hyperacute T waves in Hyperkalemia if presence of hyperkalemia was clinically known.Other differences between GM-ECG and the actual are not significant and do not lead to a potential for misinterpretation.

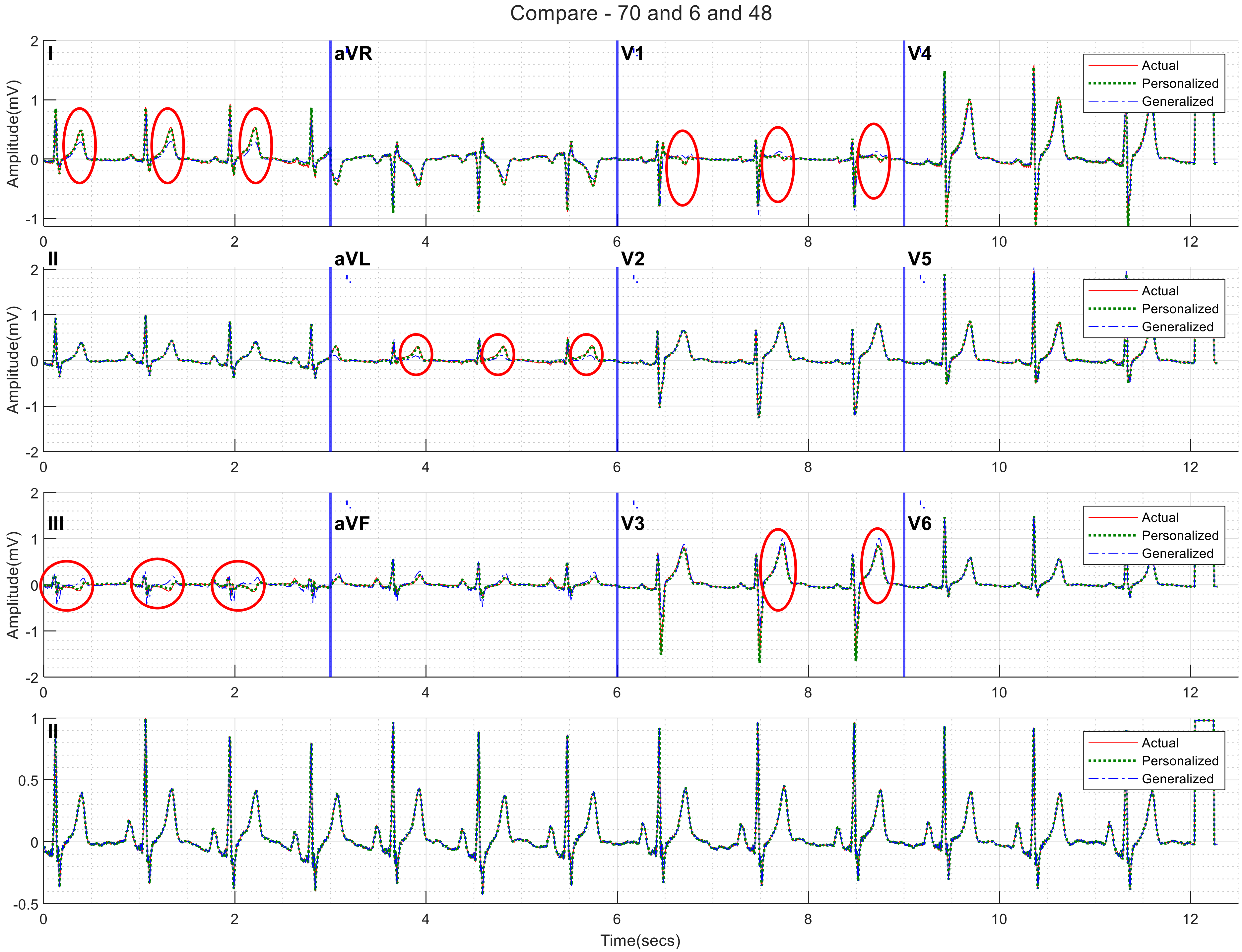


**Figure S 11. Actual diagnosis –Normal sinus rhythm; Personalized model diagnosis Sinus rhythm, tall T waves; Generalized model diagnosis – Sinus rhythm. The red markers indicate the regions in the GM- ECG that deviates from the actual and PM-ECG**

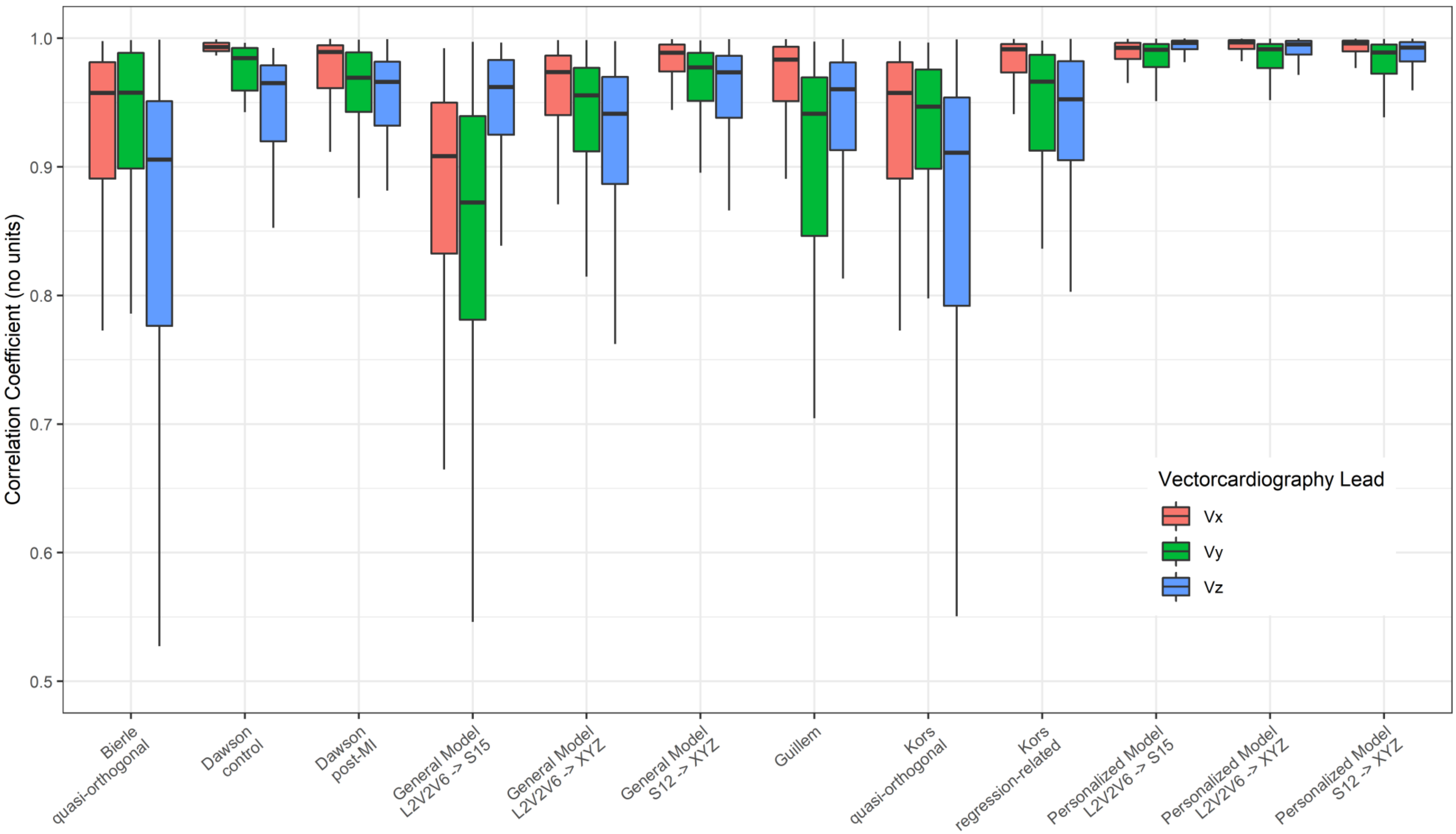


**Figure S12. Comparison of Correlation Coefficients for deriving Frank XYZ from standard 12 lead and the proposed GM-ECG and PM-ECG models**

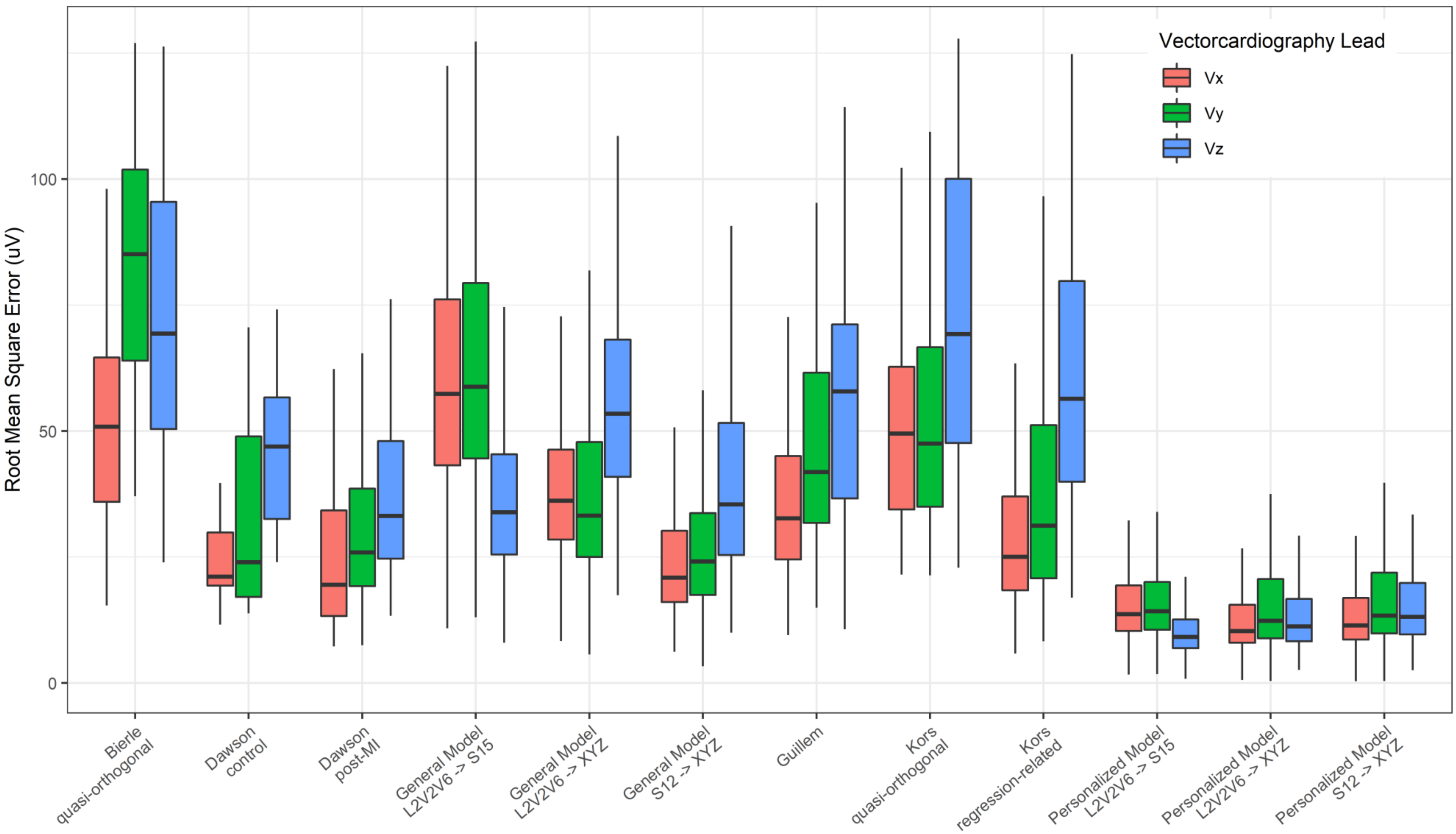


**Figure S13. Comparison of RMSE for deriving Frank XYZ from standard 12 lead and the proposed GM-ECG and PM-ECG models**

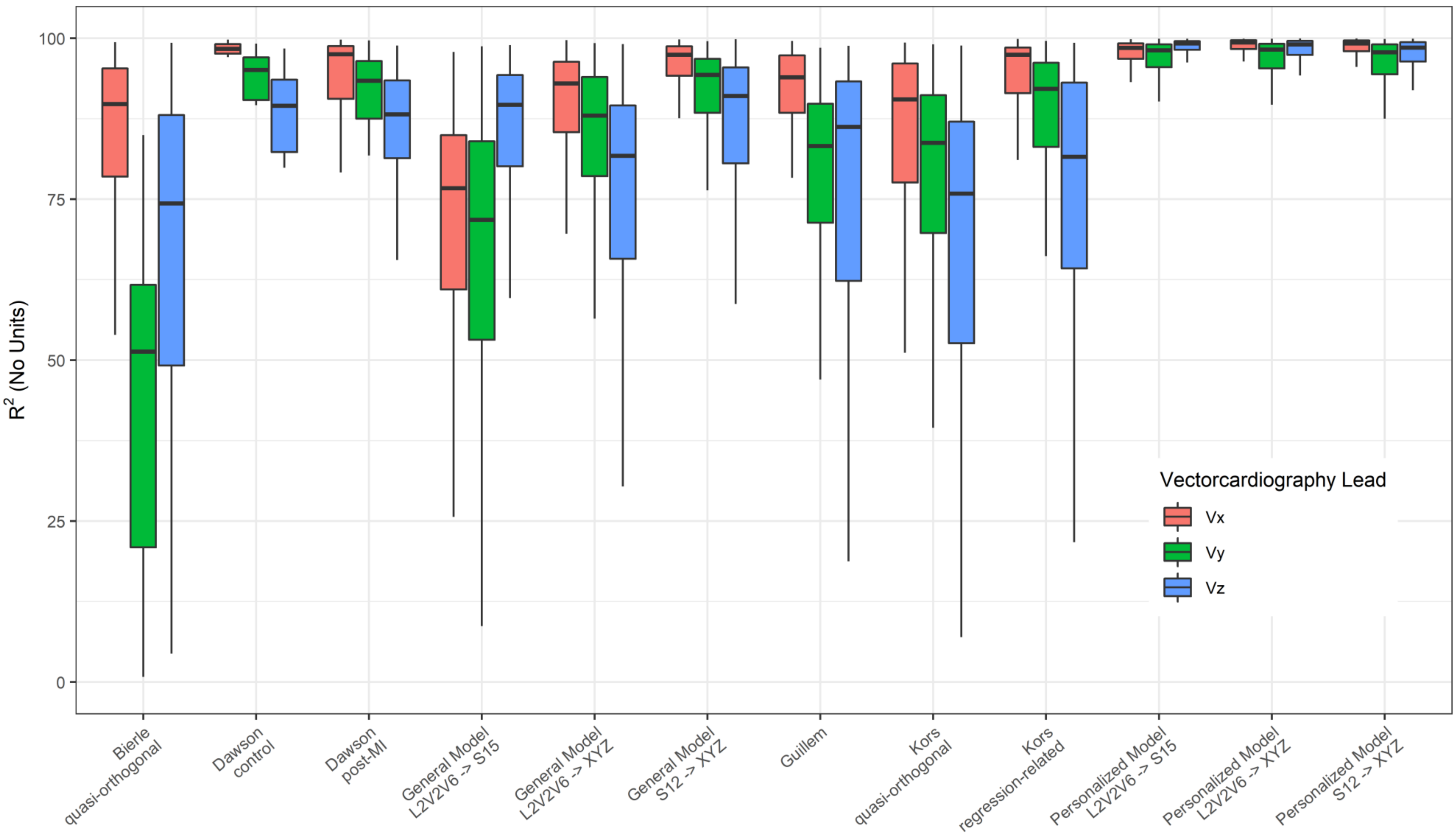


**Figure S14. Comparison of $R^2$ for deriving Frank XYZ from standard 12 lead and the proposed GM-ECG and PM-ECG models**

**Table S2. RMSE (mean ± std) for the derivation of all leads using the general and personalized Lead 2, V2, V6 to all other leads transformations**

| Method | aVF | aVL | aVR | Lead 1 | Lead 3 | V1 | V3 | V4 | V5 | Vx | Vy | Vz |
|---|---|---|---|---|---|---|---|---|---|---|---|---|
| General L2V2V6 -> S15 | **118.73 ± 92.91** | **107.18 ± 86.41** | **89.91 ± 121.76** | **74.08 ± 62.69** | **48.79 ± 48.83** | **75.53 ± 72.39** | **58.90 ± 55.13** | **50.69 ± 54.13** | **73.84 ± 75.89** | **74.13 ± 55.19** | **77.32 ± 69.04** | **42.65 ± 31.93** |
| Personalized L2V2V6 -> S15 | **30.11 ±77.84** | **30.58 ± 76.43** | **30.39 ± 136.94** | **23.79 ± 56.75** | **18.67 ± 52.81** | **23.99 ± 56.77** | **21.25 ± 43.14** | **23.11 ± 39.92** | **24.29 ± 48.77** | **23.46 ± 45.21** | **26.60 ± 70.78** | **15.54 ± 24.09** |

**Table S3. $R^2$ (mean ± std) for the derivation of all leads using the general and personalized Lead 2, V2, V6 to all other leads transformations**

| Method | aVF | aVL | aVR | Lead 1 | Lead 3 | V1 | V3 | V4 | V5 | Vx | Vy | Vz |
|---|---|---|---|---|---|---|---|---|---|---|---|---|
| General L2V2V6 -> S15 | **75.04 ± 9.54** | **86.05 ± 15.79** | **74.12 ± 33.17** | **45.52 ± 47.05** | **77.28 ± 26.60** | **83.67 ± 19.90** | **81.87 ± 28.57** | **77.56 ± 30.23** | **68.11 ± 38.59** | **62.13 ± 40.03** | **51.41 ± 47.91** | **81.78 ± 25.03** |
| Personalized L2V2V6 -> S15 | **98.18 ± 5.61** | **98.43 ± 6.87** | **96.32 ± 12.47** | **93.91 ± 9.39** | **97.02 ± 5.83** | **97.71 ± 11.32** | **97.17 ± 13.42** | **94.44 ± 9.77** | **96.19 ± 9.96** | **95.64 ± 8.37** | **94.53 ± 8.37** | **97.07 ± 7.93** |

**Table S4. Pearson Correlation Coefficient (mean ± std) for the derivation of all leads using the general and personalized Lead 2, V2, V6 to all other leads transformations**

| Method | aVF | aVL | aVR | Lead 1 | Lead 3 | V1 | V3 | V4 | V5 | Vx | Vy | Vz |
|---|---|---|---|---|---|---|---|---|---|---|---|---|
| General L2V2V6 -> S15 | **0.90 ± 0.15** | **0.95 ± 0.08** | **0.90 ± 0.16** | **0.74 ± 0.27** | **0.91 ± 0.13** | **0.94 ± 0.10** | **0.93 ± 0.14** | **0.89 ± 0.16** | **0.89 ± 0.15** | **0.84 ± 0.21** | **0.78 ± 0.24** | **0.92 ± 0.13** |
| Personalized L2V2V6 -> S15 | **0.99 ± 0.08** | **0.99 ± 0.05** | **0.98 ± 0.08** | **0.97 ± 0.05** | **0.99 ± 0.03** | **0.99 ± 0.09** | **0.98 ± 0.08** | **0.97 ± 0.07** | **0.98 ± 0.06** | **0.98 ± 0.05** | **0.97 ± 0.04** | **0.99 ± 0.04** |

**Pseudocode for Hyperparameter search using Bayesian Optimization**

**Input** ( Hyperparameter search space $\boldsymbol{\theta}$, Objective function $\boldsymbol{f(x)}$, maximum number of iterations ($\boldsymbol{n_{max}}$), acquisition function $\boldsymbol{a(x)}$)

Select initial hyperparameter configuration $\theta_0 \epsilon \theta$; $\varphi[1] = \theta_0$;

Evaluate $f(x)$ for neural network architectures defined with $\theta_0$ as hyperparameters; $y[1] = f(\theta_0)$

Obtain an initial Gaussian Process Model $Q(f|x, y)$ i.e $Q^*(f|x, y) = Q(f|\theta_0, y_0)$

**For** n=2,…,$n_{max}$

Find a new set of hyperparameters $\varphi[n]$ for evaluation that maximizes the function $a(\varphi[n])$

$y[n] = f(\varphi[n]\ )$

Update $Q(f|x, y)$ using $y[1{:}\, n], \varphi[1{:}\, n]$

**Endfor**

**Output** $\theta_{optimal} = ArgMin$[y, $\varphi$]
